# Informal food environment is associated with household vegetable purchase patterns and dietary intake in the DECIDE study: Empirical evidence from food vendor mapping in peri-urban Dar es Salaam, Tanzania

**DOI:** 10.1016/j.gfs.2020.100474

**Published:** 2021-03

**Authors:** Ramya Ambikapathi, Gerald Shively, Germana Leyna, Dominic Mosha, Ally Mangara, Crystal L. Patil, Morgan Boncyk, Savannah L. Froese, Cristiana K. Verissimo, Patrick Kazonda, Mary Mwanyika-Sando, Japhet Killewo, Nilupa S. Gunaratna

**Affiliations:** a Department of Public Health, Purdue University, USA; b Department of Agricultural Economics & International Programs in Agriculture, Purdue University, USA; c Tanzania Food and Nutrition Centre, Tanzania; d African Academy of Public Health, Tanzania; e Department of Epidemiology and Biostatistics, Muhimbili University of Health and Allied Sciences, Tanzania; f Department of Women, Children & Family Health Science, University of Illinois at Chicago, USA; g Department of Nutrition Science, Purdue University, USA

**Keywords:** Food environment, Tanzania, Diets, Food purchase patterns, Informal economy, Adults, PLHIV

## Abstract

We study the relationship between the food environment (FE) and the food purchase patterns, dietary intakes, and nutritional status of individuals in peri-urban Tanzania. In Africa, the prevailing high density of informal vendors creates challenges to characterizing the FE. We present a protocol and tool developed as part of the Diet, Environment, and Choices of positive living (DECIDE) study to measure characteristics of the FE. We mapped 6627 food vendors in a peri-urban settlement of Dar es Salaam, of which over 60% were semi-formal and informal (mobile) vendors. We compute and compare four FE metrics inspired by landscape ecology—density, dispersion, diversity, and dominance—to better understand how the informal food environment relates to food purchase patterns, diets, and nutritional status among households with persons living with human immunodeficiency virus (PLHIV).

## Highlights

• In Tanzania, a high density of informal vendors within this transitioning food system creates challenges to characterizing the food environment.
• We develop four metrics inspired by landscape ecology to characterize formal, semi-formal, and informal food environments.
• We use these metrics to evaluate associations to household food purchase patterns, energy intake, and nutritional status.
• Vegetables, especially green leafy vegetables, were primarily sold by informal mobile food vendors, most of whom were women.
• A greater density of vegetable vendors or informal vendors within 500 meters of a household is associated with a higher likelihood of vegetable purchases and lower energy intake.
• The role of informal and semi-formal food vendors in supporting consumption of nutritious diets should not be discounted, and gender sensitive actions to promote their livelihoods is needed.

## 1 Introduction

Over the past two decades, agricultural transformation and rural development in Southern and Eastern Africa have led to rapid changes in migration patterns, farm intensification, and household incomes in East Africa (AGRA, 2019; Cockx et al., 2019; FAO, 2016; Global Panel, 2017; Reardon et al., 2019). Coupled with the rise of private food enterprises and processing hubs, these changes are leading to profound shifts in food availability for consumers (Khonje and Qaim, 2019; Weatherspoon and Reardon, 2003). Where consumers acquire their food is often defined as the ‘food environment’ (FE) and can consist of multiple domains in low- and middle-income countries (LMIC) (Downs et al., 2020; Herforth and Ahmed, 2015; Turner et al., 2019). Although emerging evidence from developed countries with modern food systems provides evidence that the FE shapes diets, nutrition, and health outcomes (Caspi et al., 2012; Lytle and Sokol, 2017), the impacts of rapidly changing food environments in rapidly evolving food systems in Eastern Africa and their effects on diets, nutrition, and health outcomes remains understudied (Turner et al., 2018).

Many of the metrics used to characterize FE have been developed in high-income countries and are based on static built environments and public-use databases (Turner et al., 2018). Even these databases can be problematic. For example, a recent study from the US city of Baltimore shows that there is discordancy due to rapid changes in stores and restaurants turnovers (Caspi et al., 2012; Kharmats, 2020). Such databases are not readily available in LMICs, and they would not necessarily be applicable in contexts characterized by a predominance of informal food vendors, i.e. those with semi-permanent physical structures (umbrella, pallets, boxes, baskets), or those who are highly mobile and sell seasonally-available food (Turner et al., 2018). Research conducted in South Africa illustrates that despite the permeation of supermarkets, informal food vendors often remain the primary and most frequently used food source for the urban poor and for the food-insecure households (Battersby, 2011a, Battersby, 2011b; Battersby et al., 2016; Crush and Frayne, 2011; Crush et al., 2012; Peyton et al., 2015; Skinner, 2016; Skinner and Haysom, 2016).

These studies combined with findings from several recent reviews point to four key gaps in research on food environments and their effects on diets and nutritional status in LMIC (Downs et al., 2020; Turner et al., 2018, 2019). First, there is a need to focus on both formal (i.e. ‘built’) and informal market environments, along with self-cultivation of foods (in the ‘home garden’), and other food acquisition practices (Turner et al., 2018). Second, because most studies focus on consumption at the food group level, there is a need to examine associations between FE metrics and energy intake (Turner et al., 2019). Third, there is a need to focus on associations of FE on multiple measures of nutritional status adjusting for co-morbidities (Turner et al., 2019). Fourth, geospatial methodologies need to focus on the diversity of vendor typologies and include distance measures (Charreire et al., 2010).

We address each of these research gaps in this paper. We (1) introduce a set of FE metrics inspired by landscape ecology to characterize a food environment that includes formal, semi-formal, semi-formal, informal food vendors in peri-urban Dar es Salaam, Tanzania; (2) describe and compare a set of four FE metrics – density, dispersion, diversity (of vendor type), dominance (of vendor type) over various distances to the household; and (3) evaluate the association between these FE metrics and observed household food purchase behaviors, especially vegetable purchase patterns, diets (energy intake), and nutritional status (Body Mass Index and Waist-to-Hip ratio). Our focus is on persons living with human immunodeficiency viruses, hereafter referred as PLHIV. In this analysis, we take into account the natural food environment (cultivation of home gardens), the built food environment (formal, semi-formal, and informal food vendors), and household self-reporting of morbidity and demographics (age, gender, education, fridge ownership, assets). A key methodological innovation is that, in measuring the explanatory role of FE metrics in outcomes of interest, we explicitly account for distance to the household to capture the interaction of physical proximity on purchasing behaviors, diets, and nutrition outcomes.

## 2 Methods

### 2.1 Setting

This analysis is one of the key aims of Diet, Environment, and Choices of positive living (‘DECIDE’ study): Evaluating personal and external food environment influences on diets among PLHIV and families in Dar es Salaam, Tanzania. DECIDE study was approved by Institutional Review Boards at Purdue University and the National Institute of Medical Research in Tanzania. DECIDE is an observational cohort study set in Ukonga, a ten square kilometer peri-urban settlement outside of Dar es Salaam, bordered by the Julius K. Nyerere international airport, Tanzania-Zambia railways, military barracks, a prison, and a slaughterhouse and is nested within a larger ongoing urban surveillance system called Dar es Salaam Urban Cohort Study, hereby referred as DUCS (Leyna et al., 2017). Since 2011, DUCS has conducted annual surveillance of 21,000 households with 110,882 participants and has collected information on vital events, migration, demographics, food insecurity, and knowledge on sickle cell anemia (Leyna et al., 2017).

### 2.2 Participant recruitment

Study participants were recruited from two community-based clinics based on the following criteria between February to June 2019: 1) Adults over 18 years of age; 2) Consent to participate to both survey rounds of the study; 3) Live in the DUCS study area or close to the DUCS surveillance system; 4) Consent for a family member to participate in the study (family members were consented separately as well). We screened 2600 participants, out of whom 396 were eligible. Reasons for ineligibility were as follows: 67% did not disclose HIV status to family, 12% lived very far from the study area, 3% enrolled less than 6 months, 2% were underage, and less than 1% had missing data. Out of the 396 eligible, 7% consented to participate but moved away before the first round of interviews and 11% were not available after multiple attempts for an interview. Out of the 326 eligible at screening, 14 had missing data. Out of 312 respondents, we included 239 households that were within 1000 m of the FE surveyed area. There were no significant differences between the 73 excluded participants and the 239 included participants with regard to any of the six key outcomes described below.

### 2.3 Data collection on participants

Information on participants' age, gender, head of household status, and education was collected. At the household level data pertaining to assets, water security and access, hygiene facilities, presence of home garden (planted items were entered as free text), and food insecurity were collected. We asked about participants' household morbidity (‘Do you or does anyone in your household have the following chronic disease: hypertension, diabetes, cancer, heart diseases, other diseases?) and self-reported adherence to antiretroviral therapy (ART) in the last month. Food purchase, frequency of purchase, and location of purchase (markets, kiosks, umbrella, mobile vendor, other) in the last seven days were collected on 49 selected food items across the five food groups (staples, fresh vegetables and fruits, oils, snacks/juices, and meat). This is based on previous research conducted in Peru that was adopted and piloted for peri-urban Dar es Salam (Ambikapathi et al., 2018). The main aim of applying this food purchase tool was to examine food purchase and sourcing patterns by asking about several options within the same food group that vary by prices, so any effects of substitution due to food insecurity could be captured (Ambikapathi et al., 2018).

### 2.4 Key outcomes from the participants

We included six outcomes in this analysis: vegetable purchase in the last seven days, vegetable purchase variety in the last seven days, energy intake in kilocalories, energy adjusted for bodyweight in kilograms, Waist-to-Hip Ratio (WHR) and Body Mass Index (BMI). We defined a binary indicator of vegetable purchase if the household had purchased any of the following ten nutrient-dense vegetables in the last seven days: green peas, avocado, tomato, eggplant, amaranth leaves, spinach, pumpkin leaves, Chinese spinach leaves, carrots, and sweet potato. Vegetable purchase variety was defined as the number of different vegetables (out of the ten listed above) that was bought in the last seven days. Since this metric was among those who purchased vegetables, it had a smaller sample size of 173 households. We focus this analysis on vegetable purchase to align with the Tanzanian national nutrition guidelines for PLHIV, which explicitly promotes the consumption of vegetables, especially green leafy vegetables (Tanzania Food Nutrition Centre, 2016). Energy and dietary intake were quantified using a tablet-based 24-h recall tool, which used Tanzanian and Kenyan food composition tables to estimate intakes (Ambikapathi et al., 2019). Adult waist and hip circumference, weight, and height were measured by trained enumerators on the same day as the dietary recall was recorded. Body Mass Index (BMI) and Weight-Height Ratio (WHR) were calculated from these anthropometric measurements.

### 2.5 Data collection on food environment and classification of food environment typologies

We chose to characterize food environment metrics by vendor typologies and niches rather than food groups or food types to better target policies and governance. For example, promotion of food safety among prepared foods could be targeted to a certain typology of vendor and gender who are primarily selling a specified prepared food. It's also useful to understand how vendor niches change if a supermarket comes into a neighborhood.

**Fig. 1:**
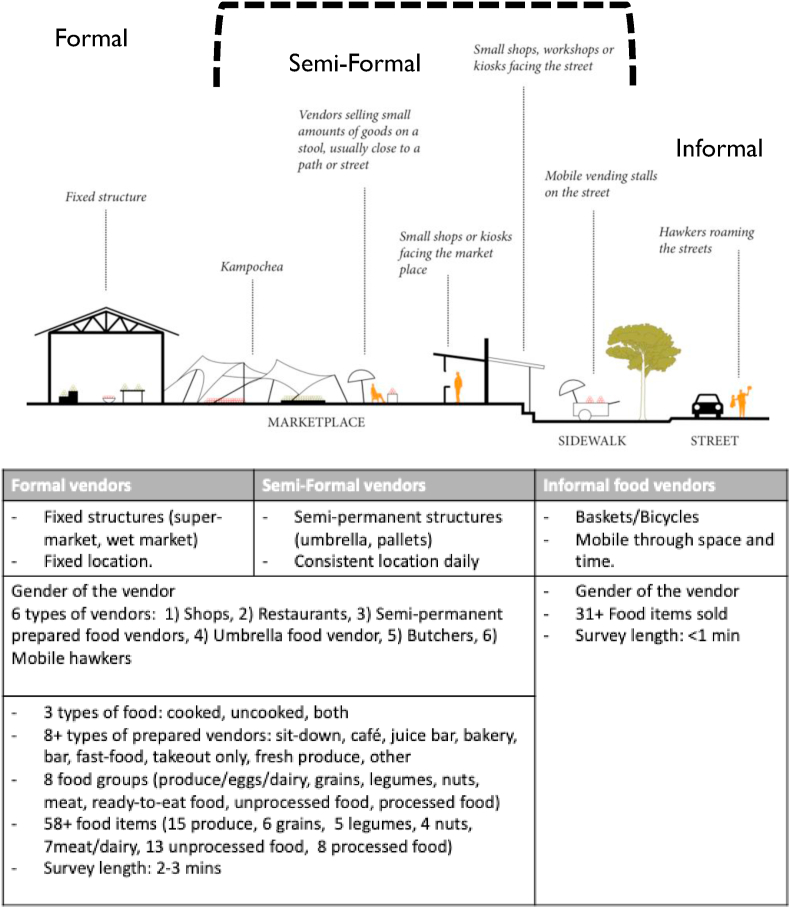
Illustration of food vendors grouped by formal, semi-formal, and informal categories in peri-urban Dar es Salaam, Tanzania. Criteria for grouping and type of information collected by vendor categories is summarized in table below. Illustration adapted from “Vendors Galore and More” (Boustedt and Mair, 2013).

The study team conducted a photo transect survey of all the different types of food vendors in the study area, and then held discussions to decide how vendors could be categorized to improve the reliability of classifications. Vendors were defined as anyone who sold perishable and non-perishable foods, prepared meals and snacks, or fresh and/or processed foods. Vendors included street hawkers, bicycle vendors, umbrella/stall/pallet/basket vendors, shops, kiosks, stores, butchers, and supermarkets. We evaluated each photo from the transect survey to define typology of vendors. Two key criteria emerged to define food vendors in this FE. These were (1) physical infrastructure, and (2) consistent daily location. Those with permanent physical infrastructure such as cement-built stores who were consistently present in the same location were tagged as formal vendors. Vendors with semi-permanent structures (e.g. wooden stalls and umbrellas) but who maintained consistent daily location were categorized as semi-formal vendors. Mobile vendors who either walked or used bicycles or carts were categorized as informal vendors. These three typologies of FE vendors are illustrated in Fig. 1 (Boustedt and Mair, 2013).

Because we wanted to conduct a full census of all vendors in the area, including vendors located in smaller streets and alleyways, we developed a tablet-based food environment survey of each vendor type (formal, semi-formal, informal) that enumerators could conduct while walking. Based on the first two criteria, skip patterns were programmed to direct to questions related to either formal and semi-formal vendors, or to informal vendors. All of the vendors were geo-tagged, including mobile vendors who were tagged where the enumerator saw them. All vendors were further characterized into six specific typologies (‘vendor typologies’): shops, restaurants, semi-formal, umbrella food vendors, butchers, and mobile hawkers. Information on gender, eight food groups, and 58 food items were collected from formal and semi-formal food vendors, based on discussions with the study team and previous literature (Battersby, 2016; Hua et al., 2014). Questions on food groups and food items also had other options for user-entered text. For informal vendors, we collected information on gender and 31 food item types sold, along with an “other” option. We also recorded the gender of the food vendor, who could have been either the owner or the employee. We kept the tool relatively short (1–2 min) because the aim was to create a field-friendly tool that captured data on all food vendors.

### 2.6 Development of FE metrics

Since households and vendors were geo-tagged, we obtained the precise distance between the two locations using the ‘geodist’ package in Stata (Picard, 2010). We calculated the FE metrics at various arbitrary buffer sizes ranging from 100 to 1000 m of the household. We focused much of the FE metrics on vendor characteristics for ease of interpretation and for targeting interventions and policy framing.

We created three categories of FE metrics inspired by landscape ecology. These metrics are typically used to describe ecological processes such as food foraging behavior or predator-prey relationships (Costanza et al., 2019; Gergel and Turner, 2017; Turner et al., 2001). Landscape ecology metrics can either address the landscape-level or the patch-level, i.e. individual small areas that make up a landscape. Similarly, we developed FE metrics at the household level (~patch-level). These categories include density, dispersion, and dominance/diversity, and are summarized in Table 1 (see below).

**Table 1:** Summary of food environment metrics.

Metric	Name	Definition	
Density	Food environment typology	Count	Informal, semi-formal, formal and all vendors
	Vegetable vendors	Count	Vendors who sell any of 10 vegetables
	Green leafy vegetable vendor	Count	Vendors who sell green leafy vegetables
**Dispersion**	Vegetable vendor hotspots/cold spots	Clusters	Vegetable vendors
	Green leafy vendor hotspots/cold spots	Clusters	Green leafy vegetable vendors
**Diversity/Dominance**	Shannon diversity of vendor typology (standardized 0 to 1)	Variety and evenness	6 vendor typology: restaurants, mobile vendors, shops, semi-formal prepared food vendors, butchers, umbrella vendors
	Dominance of vendor typology (standardized 0 to 1)	Variety and evenness	Measure of one/few vendor dominating (1- diversity). Lack of variety and evenness.

The first of these, density, is the count of vendors within the a given distance from a household. These counts can be stratified by the characteristics of the vendors; for example, number of informal or semi-formal vendors or all vendors who sell vegetables generally, or more specifically green leafy vegetables. Second, we calculated dispersion, which is a measure of spatial distribution. This identifies areas that have a higher occurrence of a specified attribute, e.g. clusters of vegetable vendors. Areas with relatively high occurrence of a specified attribute are often referred to as ‘hot-spots’ (following the example above, vegetable vendor hot-spots) and are delineated using a local spatial autocorrelation statistic called Local G* for Getis-Ord statistic (Kondo, 2016). We estimated the number of vegetable vendor hot-spots within various distances to the households. In contrast, we also estimated the number of ‘cold-spots’, i.e. areas with relatively low numbers of clusters of a specified attribute (in this case, vegetable vendors) compared to the surrounding area. Recognizing that Euclidean distance in dense urban settlements might not fully account for actual travel distances along pathways through informal settlements, we conducted sensitivity analyses on buffer size. We chose a buffer size of 300 m based on the calculated distribution of hot-spots and cold-spots, and for sensitivity analysis we compared this to a buffer size of 100 m. Conceptually, the main difference between density and dispersion of vegetable vendors is the clumping of spatial distribution; for a given density, if vendors are evenly spaced, there would be no clustering. However, if they are is a high clustering of food vendors within a buffer size of 300 m, with the same density, this area would be coded as a hot-spot.

Third, we calculated a measure of variety and evenness called diversity and dominance. We calculated standardized Shannon diversity of six types of vendor (restaurants, shops, stalls/basket vendors, umbrella vendors, mobile vendors, butchers). Shannon diversity closer to one indicates higher diversity of vendors with even distribution of number of vendors. Dominance is a measure of one type of vendor dominating the landscape, and a dominance score closer to one indicates higher relative occurrence of one type of vendor. Because the type of vendors does not change (n = 6), the mathematical relationship is dominace=(1-diversit)y; hence, it is perfectly collinear with diversity.

### 2.7 Statistical analysis

Logistic regression with robust standard errors was used to evaluate relationships between metrics on purchase of vegetables in the last 7 days (binary outcome). These results are presented as odds ratio (OR) with 95% confidence interval (CI). Multiple regression with robust standard errors was used for analysis of FE with vegetable purchase diversity, energy intake (kcal), WHR, and BMI. Two sensitivity analyses were conducted: (1) Different buffer sizes for clusters under the dispersion metrics; (2) FE metrics on energy intake adjusted for bodyweight (Kg). Descriptive statistics are presented as median and interquartile ranges (IQR). Statistical analyses were conducted in Stata, visualized in R Studio and Stata, and mapped in Geoda and QGIS.

## 3 Results

### 3.1 Food environment mapping and metrics

**Fig. 2:**
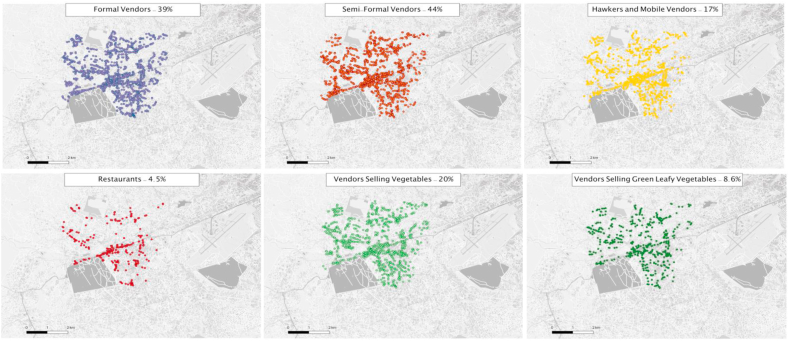
Map of food vendor types in the DECIDE study area.

**Fig. 3:**
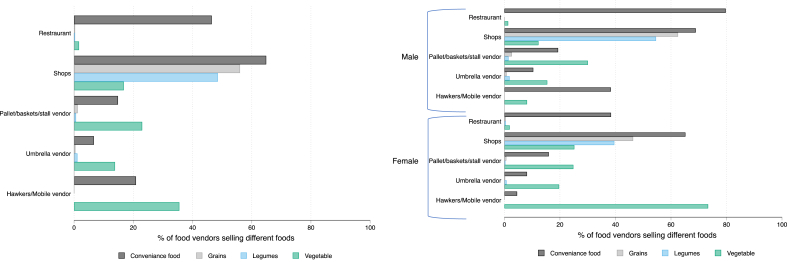
Food groups sold by type of establishment and gender of the food vendor n the DECIDE study area^1^. ^1^Gender of the food vendors had 11% data missing, with the majority missing were from semi-formal vendors 74% (n = 547 food vendors), followed by formal vendors with 15% (n = 110 food vendors), 11% (n = 86) missing informal vendors. Important to note that we recorded the gender of the main food vendor, who could have been the owner or the employee.

In Fig. 1, we summarize the tool developed in this study with an illustration from “Vendors Galore and More” (Boustedt and Mair, 2013). Using this tool, we conducted a food environment census of our study area and collected data on 6,627 vendors during March–June 2019. Table 2 gives a summary of food vendor characteristics. Shops are the predominant type of establishment (34%) in the study area, followed by umbrella vendors (23%), and basket/pallet vendors (21%), and mobile vendors (17%). Three quarters of food vendors mention that they sell food at the same place, with the highest rate of this behavior observed among formal vendors (96%) and semi-formal vendors (81%), as expected. Fig. 2 shows these vendors mapped in the study area by food environment typology, where 39% were formal vendors, 44% were semi-formal and 17% were informal vendors (mobile hawkers). Restaurants (4.5%) are mostly found along the main roads. Fig. 3 shows the four food groups sold by different vendors disaggregated by gender. Among vendors who sold vegetables (n = 1,317), 70% of the vegetables were sold by semi-formal vendors (umbrella/pallet/basket/stall) and informal vendors (hawkers/mobile vendors), and in particular, 58% vendors selling the green leafy vegetables were mobile vendors (out of 570 total vendors who sold green leafy vegetables). Shelf-stable foods such as grains, legumes, and convenience foods (wafers, biscuits, branded and unbranded chips, peanut butter, sugary drinks, candy, bread, and other snacks) were mostly sold in shops. Gender differences were observed among semi-formal food vendors and types of food sold. Seventy eight percent of the vegetable vendors were women, and over 90% of the green leafy vegetables vendors were women. It's important to note that 11% of the gender data was missing; the majority of this missing data was from semi-formal vendors (74%, n = 547 food vendors), followed by formal vendors (15%, n = 110 food vendors), and informal vendors (11%, n = 86).

**Table 2:** Characteristics of the food environment census of 6627 food vendors in peri-urban Dar es Salam, Tanzania, 2019.

	Formal	Semi-formal	Informal	Total
	N = 2590	N = 2917	N = 1120	N = 6627
Gender of vendor^1^				
Male	55.9 (1387)	30.0 (712)	53.5 (553)	45.1 (2652)
Female	44.1 (1093)	70.0 (1658)	46.5 (481)	54.9 (3232)
				
Does the food vendor sell at the same place throughout the day?				
No	3.9 (102)	18.8 (547)	92.3 (1034)	25.4 (1683)
Yes	96.1 (2488)	81.2 (2370)	7.7 (86)	74.6 (4944)
				
Type of establishment				
Restaurant	11.6 (301)	0.0 (0)	0.0 (0)	4.5 (301)
Shop	88.1 (2281)	0.0 (0)	0.0 (0)	34.4 (2281)
Pallet/baskets/stall	0.0 (0)	47.7 (1391)	0.0 (0)	21.0 (1391)
Umbrella vendors	0.0 (0)	52.3 (1526)	0.0 (0)	23.0 (1526)
Hawker/mobile	0.0 (0)	0.0 (0)	100.0 (1120)	16.9 (1120)
Butcher	0.3 (8)	0.0 (0)	0.0 (0)	0.1 (8)
				
Type of food sold				
Prepared or cooked	14.4 (374)	53.5 (1560)	26.8 (300)	33.7 (2234)
Uncooked or raw products	69.7 (1805)	44.7 (1304)	73.1 (819)	59.3 (3928)
Both prepared and uncooked	15.9 (411)	1.8 (53)	0.1 (1)	7.0 (465)
				
Type of prepared food				
Sit down	6.8 (176)	7.7 (224)	8.8 (98)	7.5 (498)
Take out	63.1 (1635)	62.6 (1826)	78.0 (874)	65.4 (4335)
Fast food	0.1 (2)	0.1 (4)	0.4 (4)	0.2 (10)
Café	1.5 (40)	0.4 (12)	0.0 (0)	0.8 (52)
Bakery	0.4 (11)	0.0 (1)	0.0 (0)	0.2 (12)
Bar	0.7 (17)	0.0 (0)	0.0 (0)	0.3 (17)
Juice bar	0.1 (2)	0.2 (7)	0.0 (0)	0.1 (9)
Fresh vegetables and fruits	0.2 (6)	2.8 (83)	9.2 (103)	2.9 (192)
Other	27.1 (701)	26.1 (760)	3.7 (41)	22.7 (1502)
				
Sells vegetables^2^	29.4 (388)	40.3% (531)	30.2 (398)	100% (1317)
Sells green leafy vegetables^2^	3.3 (86)	26.8% (153)	58.0 (331)	100% (570)
				

^1^Gender of the food vendors had 11% data missing, with the majority missing were from semi-formal vendors 74% (n = 547 food vendors), followed by formal vendors with 15% (n = 110 food vendors), 11% (n = 86) missing informal vendors. important to note that we recorded the gender of the main food vendor, who could have been the owner or the employee. ^2^These results are presented as proportion across columns.

Table 3 shows the summary of four metrics by distance to the household. There is high food vendor density within close proximity of these sampled households. The median number of food vendors within a 100-m buffer distance is 11 (IQR: 4, 15); this number grows to 812 food vendors (IQR: 612, 998) when the buffer distance is increased to 1000 m. In other words, 50% of the households have at least 812 food vendors within 1 km (0.6 miles) of their household. Composition of informal, semi-formal, and formal vendors remains relatively constant at various distances, with semi-formal and informal vendors making up to 57% of the vendors across different distances to the household. There is greater access to vegetable vendor hot-spots and cold-spots at 500 m onwards. Diversity of vendors is homogeneous across different distances to the household, and similarly, dominance indicators increase slightly from 0.05 at 100 m to 0.17 at 1000 m. Pearson correlation of FE metrics within households is shown in Supplement Figure 1. All FE metrics are positively correlated, except for an interesting correlation pattern that emerged with 1) dominance and informal food vendors density, and 2) dominance and vegetable cold-spot clusters (see Supplement Figure 1).

**Table 3:** Food environment metrics by distance to the household.

		Distance to household					
Name	Metric	100 m	200 m	300 m	500 m	700 m	1000 m
All vendors (n)	Density	11 (4,15)	37 (24,50)	80 (58,125)	244 (163,296)	492 (343,540)	812 (612,998)
Informal vendors (n)	Density	1 (0,3)	5 (3,10)	13 (6,26)	39 (27,54)	86 (56,101)	150 (106,215)
Semiformal vendors (n)	Density	4 (1,6)	16 (8,24)	32 (21,47)	92 (67,121)	186 (135,224)	298 (226,390)
Formal vendors (n)	Density	5 (1,9)	16 (9,20)	35 (24,52)	102 (68,124)	210 (141,225)	336 (263,421)
Veg density vendors(n)	Density	1 (0,3)	6 (4,10)	16 (12,25)	46 (35,59)	88 (68,104)	153 (126,190)
Green.veg density vendors (n)	Density	0 (0,1)	2 (1,4)	6 (3,11)	18 (15,26)	38 (29,49)	69 (57,88)
Veg. hotspot clusters (0.1 km buffer) (n)	Dispersion	0 (0,0)	0 (0,1)	1 (0,3)	3 (2,10)	13 (10,24)	34 (24,39)
Veg. cold-spot clusters (0.1 km buffer) (n)	Dispersion	0 (0,0)	0 (0,0)	0 (0,1)	0 (0,111)	2 (0,166)	14 (0,166)
Veg. hotspot clusters (0.3 km buffer) (n)	Dispersion	0 (0,0)	0 (0,0)	0 (0,1)	2 (0,11)	16 (2,26)	28 (13,66)
Veg. cold-spot clusters (0.3 km buffer) (n)	Dispersion	0 (0,0)	0 (0,2)	0 (0,4)	2 (0,162)	2 (1264)	61 (2312)
Dominance of vendor typology (standardized 0 to 1)	Dominance	0.1 (0.1,0.2)	0.1 (0.1,0.2)	0.1 (0.1,0.2)	0.1 (0.1,0.2)	0.2 (0.1,0.2)	0.2 (0.2,0.2)
Variety of 6 vendor typology (standardized 0 to 1)	Diversity	0.9 (0.8,0.9)	0.9 (0.8,0.9)	0.9 (0.8,0.9)	0.9 (0.8,0.9)	0.8 (0.8,0.9)	0.8 (0.8,0.8)

Results presented in median [IQR].

### 3.2 Demographics of DECIDE participants

**Fig. 4:**
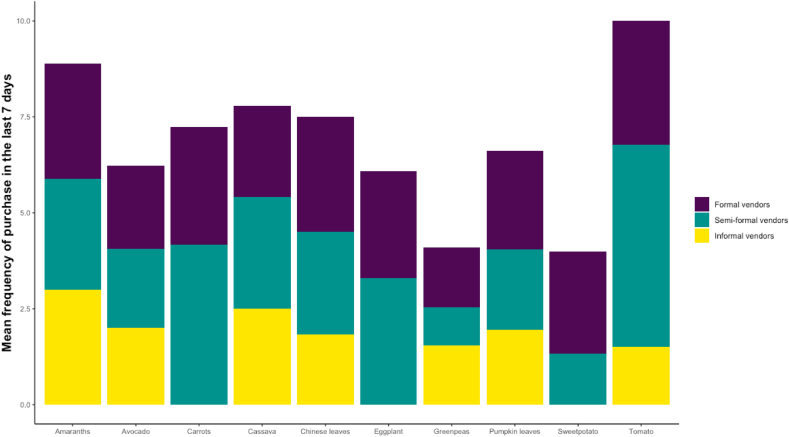
Household food sourcing survey: purchase frequency of vegetables in the last 7 days by formal, informal, and semi-formal food vendors.

Demographics and key outcomes of the participants are summarized in Table 4. Although the prevalence of any food insecurity is high at 60%, this is comparable to the surveillance conducted in the same setting among the general population (Leyna et al., 2017). A third of the participants reported cultivation of home gardens and the most commonly grown plants included sweet potato leaves and cassava leaves. Seventy-two percent of households purchased vegetables in the last week, and there are significant gender differences in reporting, possibly due to gender roles in household food purchasing. Women report higher diversity of vegetable purchase (4 vegetables, tomato, carrots, pumpkin leaves, spinach) compared to men who report a median of two vegetables purchased in the last 7 days (tomato, amaranth). Household food sourcing and purchase frequency patterns of vegetables indicate that these are primarily purchased from semi-formal and informal vendors, affirming the composition and characteristics of vendors found in the FE (see Fig. 4).

**Table 4:** Household demographics, individual characteristics, and key outcomes of DECIDE participants.

Household Characteristics	Male	Female	Total	p-value
	N = 61	N = 178	N = 239	
Food Security (HFIAS scale)				0.65
Food secure	32.8 (20)	27.5 (49)	28.9 (69)	
Mildly FIA	6.6 (4)	11.8 (21)	10.5 (25)	
Moderate FIA	29.5 (18)	28.7 (51)	28.9 (69)	
Severe FIA	31.1 (19)	32.0 (57)	31.8 (76)	
# of households that share toilet facilities with participant				0.62
None	47.5 (29)	43.8 (78)	44.8 (107)	
One	8.2 (5)	14.0 (25)	12.6 (30)	
Two to five	36.1 (22)	32.0 (57)	33.1 (79)	
More than five	8.2 (5)	10.1 (18)	9.6 (23)	
Dwelling rented from someone not living in the household				0.016
Yes	27.9 (17)	45.5 (81)	41.0 (98)	
No	72.1 (44)	54.5 (97)	59.0 (141)	
Cultivation of home garden	31.1 (19)	27.5 (49)	28.5 (68)	0.59
Refrigerator access (%)	31.1 (19)	31.5 (56)	31.4 (75)	0.96
Household with co-morbidity	21.3 (13)	31.5 (56)	28.9 (69)	0.13
				
**Individual Characteristics**				
Age (years)	44 (38,50)	39 (31,46)	40 (33,47)	<0.001
Education with grade 7 or above	90.2 (55)	77.0 (137)	80.3 (192)	0.025
Current marital status				0.72
Never married	16.4 (10)	17.4 (31)	17.2 (41)	
Currently married or living with partner	52.5 (32)	44.4 (79)	46.4 (111)	
Separated	16.4 (10)	15.7 (28)	15.9 (38)	
Divorced	4.9 (3)	6.2 (11)	5.9 (14)	
Years since HIV diagnosis	3 (1,6)	4 (2,8)	4 (2,8)	0.079
Self-reported adherence to ARVs over the last month				0.53
Poor	0.0 (0)	3.4 (6)	2.5 (6)	
Fair	6.6 (4)	6.2 (11)	6.3 (15)	
Good	60.7 (37)	56.7 (101)	57.7 (138)	
Very good	32.8 (20)	33.7 (60)	33.5 (80)	
				
**Key outcomes**				
Purchased (any 10) vegetables in the last 7 days	52.5 (32)	79.2 (141)	72.4 (173)	<0.001
Vegetable purchase diversity (among those who purchased)	2.0 (1.0,4.5)	4.0 (3.0,6.0)	4.0 (2.0,5.0)	<0.001
Energy intake (Kcal)	3105.6 (2308.4,4422.0)	2534.7 (1640.3,3445.3)	2673.2 (1775.6,3646.6)	0.004
Energy (kcal)/weight (kg)	51.6 (33.5,66.6)	44.9 (31.4,59.3)	46.4 (31.8,62.6)	0.21
Body Mass Index (kg/m2)	22.4 (20.3,25.5)	23.7 (20.7,27.7)	23.2 (20.7,27.2)	0.043
Underweight (BMI<18.5)	9.8 (6)	9.6 (17)	9.6 (23)	0.95
Overweight/Obese (BMI>25)	24.6 (15)	39.9 (71)	36.0 (86)	0.032
Waist-to-hip ratio	0.9 (0.8,0.9)	0.8 (0.8,0.9)	0.8 (0.8,0.9)	0.015

A typical participant in the DECIDE household was a woman who was 39 years of age (IQR: 31,46) and had been diagnosed with HIV for four years. Over 90% of the participants report good adherence to their ART for HIV and over a third reported co-morbidity of chronic diseases in the household (either themseles or someone in the family). DECIDE study shows evidence of nutrition transition, where 10% of the adults are underweight and 35% are overweight or obese, and these do vary significantly by gender. Gender differences in all of the key outcomes are significant except for energy (kilocalories) adjusted for bodyweight.

### 3.3 Density FE metrics on household outcomes

**Fig. 5:**
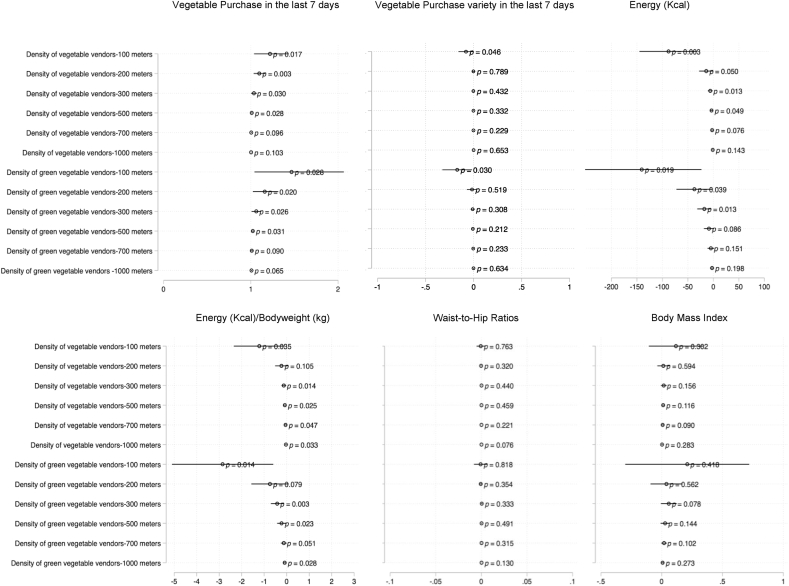
Results from multivariate regression models on 6 key outcomes: panels shows odds ratio of vegetable purchase in the last 7 days, linear regression results on vegetable purchase variety, energy intake (kilocalories), energy adjusted for bodyweight, waist-to-hip ratios and body mass index (kcal). All models adjusted for head of household status, asset quartiles, gender, age, house ownership, years since HIV diagnosis, education, presence of home garden and fridge.

Bivariate associations between FE metrics on outcomes are shown as scatter plots with lowess and linear fit in Supplemental Figs. 2–5. Results from the multivariate model are shown in Fig. 4, which adjusted for head of household status, asset quartiles, gender, age, education, house ownership, years since HIV diagnosis, presence of home garden and fridge ownership. We observe that a greater density of vegetable vendors within 500 m of home increases the likelihood of purchasing vegetables (p < 0.028) in the last week, and that this effect increases in a dose-response manner with lower buffer distances. For example, households that have greater density of vegetable vendors within 100 m are more likely to have bought vegetables in the last week (OR: 1.2 [95%CI: 1.03,1.4]). We observed similar trends with association for green leafy vegetable vendor density within 100 m on vegetable purchase (OR: 1.5 [95%CI: 1.04, 2.1]). These dose-response associations also show consistent pattern with energy intake. Greater density of vegetable vendors within 100 and 200 m of the household was associated with lower total energy intake of −88.0 Kcal [95%CI: −145.1, −31.0] and −14.0 Kcal [95%CI: −27.9, −0.1], respectively. Similarly, greater green leafy vegetable vendor density within 100 and 200 m of the household was associated with lower total energy intake of −140.6 Kcal [95%CI: −257.8, −23.4] and −37.2 Kcal [95%CI: −72.6, −1.9], respectively. These associations were consistent even after adjusting for bodyweight (see bottom panel in Fig. 5).

**Fig. 6:**
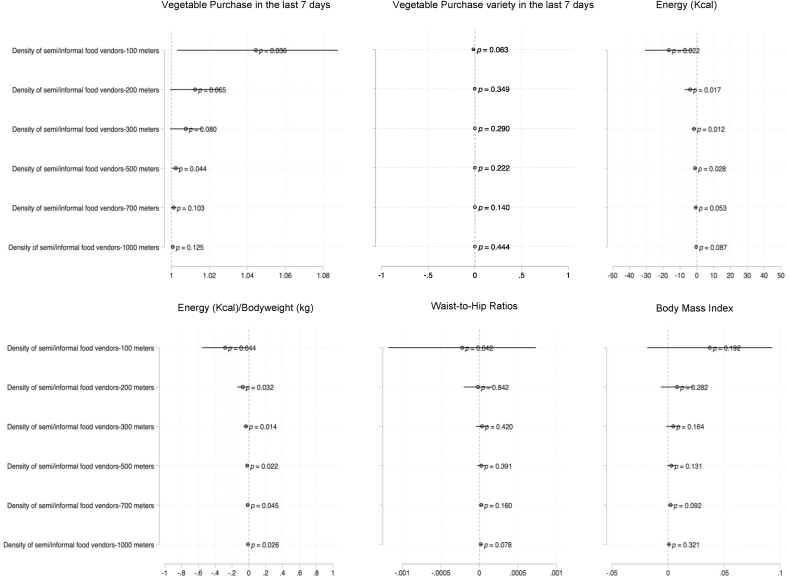
Results from multivariate regression models on 6 key outcomes: panels shows odds ratio of vegetable purchase in the last 7 days, linear regression results on vegetable purchase variety, energy intake (kilocalories), energy adjusted for bodyweight, waist-to-hip ratios and body mass index (kcal). All models adjusted for head of household status, assert quartiles, gender, age, house ownership, years since HIV diagnosis, education, presence of home garden and fridge.

However, neither the density of vegetable vendors nor the density of green leafy vegetable vendors was associated with vegetable purchase variety in the last 7 days. We also do not see any associations of vegetable and green leafy vegetable density on waist-to-hip ratios or body mass index (see Fig. 5). These trends were observed when we examine associations of density of semi-formal and informal vendors on the same six outcomes (see Fig. 6).

### 3.4 Dispersion FE metrics on household outcomes

Results of dispersion metrics, hot-spots and cold-spots of vegetable vendors with two buffers sizes (100 m and 300 m) are shown in Supplemental Figs. 6–8. As expected, we do not see any association of dispersion metrics with any of the outcomes at 100 m distance from the household, because almost no households had vegetable hot-spots or cold-spots (see Table 3) within 100 m, regardless of the buffer sizes. Vegetable vendor hot-spots with 100 m buffer size, at a distance of 200 and 300 m within households are significantly associated with higher likelihood of vegetable purchase in the last seven days with OR of 1.15 [95%CI: 1.01, 1.31] and OR of 1.09 [95%CI: 1.00,1.17], respectively. However, these associations do not hold with the larger buffer size of 300 m. Finally, we do not see any robust associations of dispersion of vegetable vendors, either hot-spots or cold-spots at two buffer sizes, with energy intake, energy adjusted for bodyweight, WHR, or BMI (see Supplemental Figs. 7 and 8).

### 3.5 Dominance FE metrics on household outcomes

Since dominance and diversity were perfectly collinear, we present results for dominance metrics of six food establishment types on household outcomes in Supplemental Fig. 9. Lower dominance of one type of food vendors at 100 m is associated with lower waist-to-hip ratios (p-value<0.032), while higher dominance at 1000 m is associated with higher WHR (p-value<0.027). Because there is no dose-response relationship with distance and the same metric, it is likely that this is a false-positive.

Overall, we do not see any robust associations of dominance and diversity of food vendor establishment on any of the 6 dietary and nutrition outcomes. The bivariate associations between dominance metrics and nutritional status (see Supplemental Figs. 4 and 5) do not show clear linear associations, and there were no variations in both exposures and outcomes so that might explain the lack of associations.

Across all metrics, belonging to higher asset quartiles and being a woman is significantly associated with higher BMI, while presence of home garden (reduces), age (increases), household renting (increases), are significantly associated with WHR. Cultivation of home garden does not affect vegetable purchase patterns, vegetable purchase variety, or energy intake in the adjusted models. Implications of these findings are discussed below.

## 4 Discussion

Ecological concepts and methods have long been borrowed and used in epidemiological studies (Earn et al., 1998; Kitron, 1998). The phrase “food environment” itself comes from ecology to describe food and nutrition availability for herbivores (Downs et al., 2020). In this analysis, inspired by landscape ecology, we developed methodology, tool, and metrics (density, dispersion, and dominance) to characterize and quantify a dynamic urban foodscape typically found in LMIC context, with a high prevalence of semi-formal and informal mobile vendors. Cross-sectional evaluation of these FE metrics on household food purchase, diets and nutrition outcomes revealed two important findings: 1) Higher density of vegetable vendors and green leafy vegetable vendors within 500 m of the household is associated with higher odds of vegetables purchase and lower total energy intake by 14–88 kilocalories; 2) these associations show a dose-response relationship where the closer the vegetable vendor density is to the household, greater the magnitude of the effect, *i.e.* higher odds of vegetable purchase and lower total calorie intake. One of the advantages of focusing metrics on food vendor typology (as opposed to vendors by food groups as previous studies have done) is that it provides a framework for describing and assessing changes in food environment that may occur as supermarkets move into poor urban neighborhoods. For example, supermarkets may fill in the niche of providing staples and shelf-stable foods, which might change the competitiveness and density of other types of food vendors such as shops or stores (Battersby and Crush, 2014). Although in this analysis we do not see associations of dispersion of vegetables food vendors and diversity of food vendor on our key outcomes, context is undoubtedly important, and research should continue to explore these concepts in a range of settings, focusing on food group or food item-level availability. The utility of using landscape ecology metrics to describe food environment in not limited to LMIC setting: a recent study conducted in Cleveland, Ohio, USA illustrates the use of diversity metrics on census data, classifying foodscapes as swamp, desert, island, and trophic, and shows the changes in the ratios of these four geometric foodscapes over time (Pike Moore, 2020).

In this analysis, 28% of the participants reported cultivation of home garden that typically consisted of vegetables, and particularly green leafy vegetables. However, we do not see any significant association of home garden with diets or the purchase of vegetables in the adjusted models. We posit two possible explanations. First, participants who grow vegetables typically are those who own their own house, thus belonging to a higher wealth stratum than those who do not grow vegetables. Accounting for home ownership and assets in the adjusted models likely masks any additional importance of home gardens. Second, it remains possible the home garden does not provide a sufficient and stable source of vegetables. In a separate analysis of the qualitative research in the DECIDE study, we find that water insecurity (along with other inputs) is a key limiting factor for home gardening. Although Tanzania does have a political history of enabling urban agriculture, this is limited by water access and agricultural inputs (Crush et al., 2011; Dongus et al., 2009). Cultivation of a home garden does not directly relate to frequent sourcing from own cultivation (Crush et al., 2011). For example, the African Food Security Urban Survey (AFSUN) conducted in 2008–09 shows that cultivation of home gardens is as high as 64% in Blantyre, Malawi but over half of these households mention they only source food once a year from their home garden, and looking across 11 cities in nine countries concludes that urban agriculture appears to contribute only modestly at present to the diets of food-insecure, low-income urban populations (Crush et al., 2011).

From our household food sourcing survey, we noted that participants primarily sourced vegetables from semi-formal/informal vendors (see Fig. 4). This corroborates the food environment census data, where we find that over 70% of the vegetables were sold through semi-formal/informal vendors. Gender disaggregation of these vendors revealed that 78% are women; even more so with green leafy vegetables, of which 95% are sold by women. These findings are similar to those of a vegetable supply chain study conducted by Fischer and colleagues in Tanzania (Fischer et al., 2018). They find that women primarily sold directly to the consumer, as opposed to men who primarily sold to restaurants and supermarkets (Fischer et al., 2018). They also show that women received the profits made through the sales of certain vegetables such as amaranth leaves, onions, and Ethiopian mustard, while men mainly received the profits from maize, sorghum, and pigeon pea sales (Fischer et al., 2018), emphasizing that vegetable food vending is a common source of livelihood for women in these communities. This ties into the larger countrywide and regional trend of women contributing more to the informal economy than men (76% in Tanzania and 74% sub-Saharan Africa) (Skinner, 2016). Thus, any policy promoting vegetable food vendors needs to be gender-sensitive.

Tanzania is part of the second wave of supermarket diffusion that first took place in South Africa and Kenya in the mid-2000's (Weatherspoon and Reardon, 2003). Despite these changes, we found semi-formal and informal vendors are the main source of most foods, including fresh produce, in poor urban food-insecure communities. These results are consistent with the studies done in low-income urban populations in Eastern and Southern Africa. The AFSUN survey showed that 70% of households acquired food through the informal food environment, and with higher frequency of interaction (daily) compared to the supermarkets which were only frequented once a month (Skinner and Haysom, 2016). A more recent ethnography study in Blantyre, Malawi suggests that even among middle-class households in urban environments, the majority of the food is sourced in the informal market—accessed via domestic helpers—and that supermarkets are accessed less frequently and mostly for shelf-stable bulk goods, such as oil and sugar (Riley, 2019).

This leads to an important discussion on definitions of semi-formality and informality of food vendors. We developed these definitions based on discussions with the study team who reside in the study area. The main distinction between semi-formal and informal food vendors is consistent presence. For example, an umbrella vendor in front of a formal shop may not have a formal license to operate but may pay the shop owner for space and security purposes (Ahmed et al., 2019). Riley's ethnography research in Malawi on various food vendor relationships in markets (municipal, designated, unsanctioned, traditional peri-urban) resonates with a similar concept where food vendors straddle between formality and informality in terms of space and business (Riley, 2019). If we use the definition outlined by Downs et al. where they define informal as the lack of regulatory governance, some of the formal vendors assessed in our study might be classified as informal (Downs et al., 2020). Moreover, Downs et al. classify wet markets as informal whereas Werthiem-Heck and Raneri classify wet markets as formal in their food environment research in Vietnam (Downs et al., 2020; Wertheim-Heck and Raneri, 2019). While standardized definitions are useful for global comparisons, contextual definitions might aid better in local policy framing and targeting. For example, informal vendors are re-sellers who often trade in goods that they themselves purchase in formal markets. They then either break down quantities into smaller conveniently consumable volumes or move the goods closer to final consumers as a way of providing convenience. When thinking about policy, some of the policies that might be levied on the formal sector (e.g. taxes) may in turn be passed through to informal vendors, so policy makers need to differentiate on this basis and avoid situations where they might double-tax something by attempting to tax the formal sector and the informal sector.

There are several limitations to this study that may limit its interpretation. First, as a cross-sectional survey, the associations between FE metrics and household outcomes should not be assumed as causal. However, we do observe dose-response relationship with proximity and magnitude, and establish plausible mechanisms from food environment to diets through food purchasing patterns. Second, as with any use of landscape metrics there is a boundary effect, i.e. households at the edge of the surveyed area might have distorted FE exposure. We did a visual check on the locations of the included households (n = 239) with the larger food environment census map to ensure there are no households on the boundary. Additionally, natural and built boundaries occur in our study area, such as army barracks in the west and the railway track in the south of the study area (see Fig. 2). Third, like any other cross-sectional study, mobile vendors were geotagged at the location they were observed, so there might be spatial and temporal variability of mobile vendors that could affect the association with the outcomes. We did a second round of data collection on a subset of the study area to assess within day and weekday spatial and temporal variability of these vendors, and in a forthcoming analysis, we address these natural variabilities as well as provide a metric for capturing these changing food environments. Fourth, caution should be exercised in generalizing these effects on outcomes because this is a special population with a higher burden of chronic diseases. We do note that household demographics and nutrition outcomes in the larger DUCS surveillance systems are comparable to this sample, thus, we highlight that these associations might be only relevant among other East African low-income urban food-insecure populations with high chronic disease burden. Fifth, we did not collect information on religion or tribal affiliation as mandated by local IRBs, and these factors may affect diets and nutritional status. Finally, both a limitation and a lesson learned, is that we had 11% missing data on the gender of food vendors. There was confusion over ownership of the business versus the person vending for their own livelihood, and another confusion arose when there were multiple people vending for the same food-vending business.

To our knowledge, our study is the first to quantify the association of both formal and informal FE on household purchases, diets, and nutritional status within the same population. Here, we have developed methodology, tool, and metrics for other researchers to replicate the findings (protocol and tools are available upon request). We have planned several future analyses, first examining the FE on household food security and consequently on food purchase and dietary adequacy, and second, assessing food environment variability.

Our study adds to several research endeavors that have mapped informal food environments in Southern and East Africa, notably the AFSUN survey, where extensive research on food environment was conducted among poor urban communities in Cape Town, South Africa, and community based participatory research combined with innovative balloon mapping was conducted among urban informal settlements in Nairobi, Kenya (Ahmed et al., 2019; Battersby et al., 2016). There are important lessons learned in terms of urban food insecurity research, and policy from these studies as these were the first two countries that were part of the first wave of supermarket diffusion in Africa; these lessons learned could be extended to Tanzania, Uganda, Ghana, Nigeria, Democratic Republic of the Congo as the second wave of supermarket diffusions settles in these countries (Crush and Frayne, 2011; Peyton et al., 2015; Skinner, 2016; Skinner and Haysom, 2016; Weatherspoon and Reardon, 2003). A common theme in these studies is the lack of visibility of semi-formal and informal vendors in the urban nutrition policy agenda (Skinner, 2016; Skinner and Haysom, 2016). Semi-formal/Informal food vending is a significant source of livelihood for the urban poor, especially for women, and additionally, operationalizes the convenience dimension of food environment for the consumers (Tacoli, 2016). The role of semi-formal/informal vendors in promoting nutritious (and non-nutritious) food could be harnessed in public health nutrition programs, along with the gender-sensitive promotion of livelihoods.

## Authorship

R.A. designed the tools, developed the methods, led the analysis, and wrote the manuscript with input from all co-authors; R.A., N·S.G., M.M.S., JK, GL, led the implementation of the study that provided data for these analyses, D.M., A.M, led the fieldwork and data collection; R.A. and N·S.G. have the primary responsibility for final content. All authors read and approved the final manuscript. This paper was presented at the Agriculture Nutrition Health (ANH) conference in July 2020.

## Funding sources

Diet, Environment, and Choices of positive living (DECIDE study): Evaluating personal and external food environment influences on diets among PLHIV and families in Dar es Salaam, Tanzania study has been funded by the Drivers of Food Choice (DFC) Competitive Grants Program, which is funded by the UK Foreign, Commonwealth and Development Office (formerly known as DFID) and the Bill & Melinda Gates Foundation (ID: OPP1110043), and managed by the University of South Carolina, Arnold School of Public Health, USA; Shively acknowledges the support provided by the Feed the Future Innovation Lab for Nutrition, which is funded by the United States Agency for International Development; however, the views expressed do not necessarily reflect those of the sponsoring agencies or the UK or US Government's official policies.

## Declaration of competing interest

The authors declare that they have no known competing financial interests or personal relationships that could have appeared to influence the work reported in this paper.
