# Supplementary material for: Informal food environment is associated with household vegetable purchase patterns and dietary intake in the DECIDE study: Empirical evidence from food vendor mapping in peri-urban Dar es Salaam, Tanzania
Source: Glob Food Sec. 2021 Mar;28:100474. doi: 10.1016/j.gfs.2020.100474 (PMC7938223; doi:10.1016/j.gfs.2020.100474)

**Supplement tables and Figures**:

Supplemental Figure 1 Correlation matrix of all food environment metrics at various distances within household (in meters).


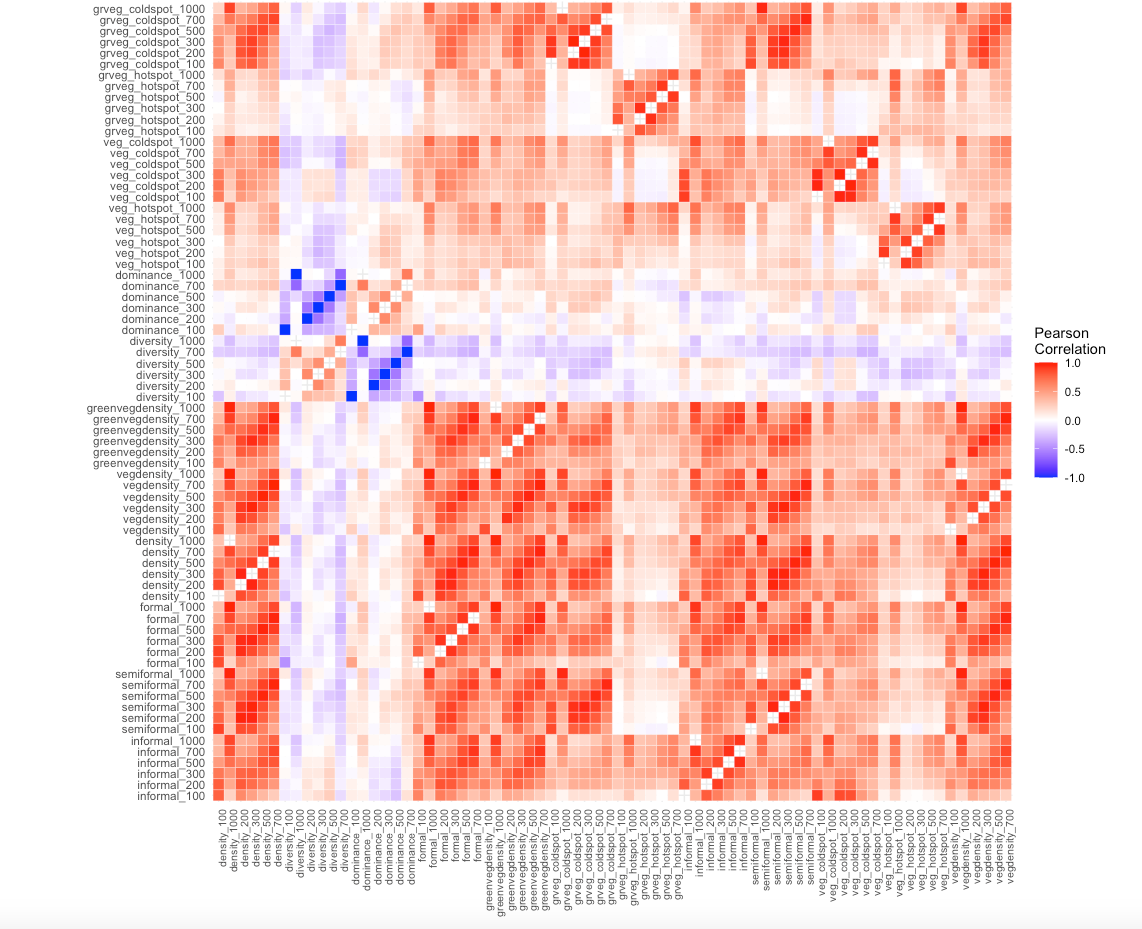


Supplemental Figure 2: Scatter plot with linear and lowess fit of various food environment metrics on vegetable purchase diversity

Supplemental Figure 3: Scatter plot with linear and lowess fit of various food environment metrics on energy (kcal)


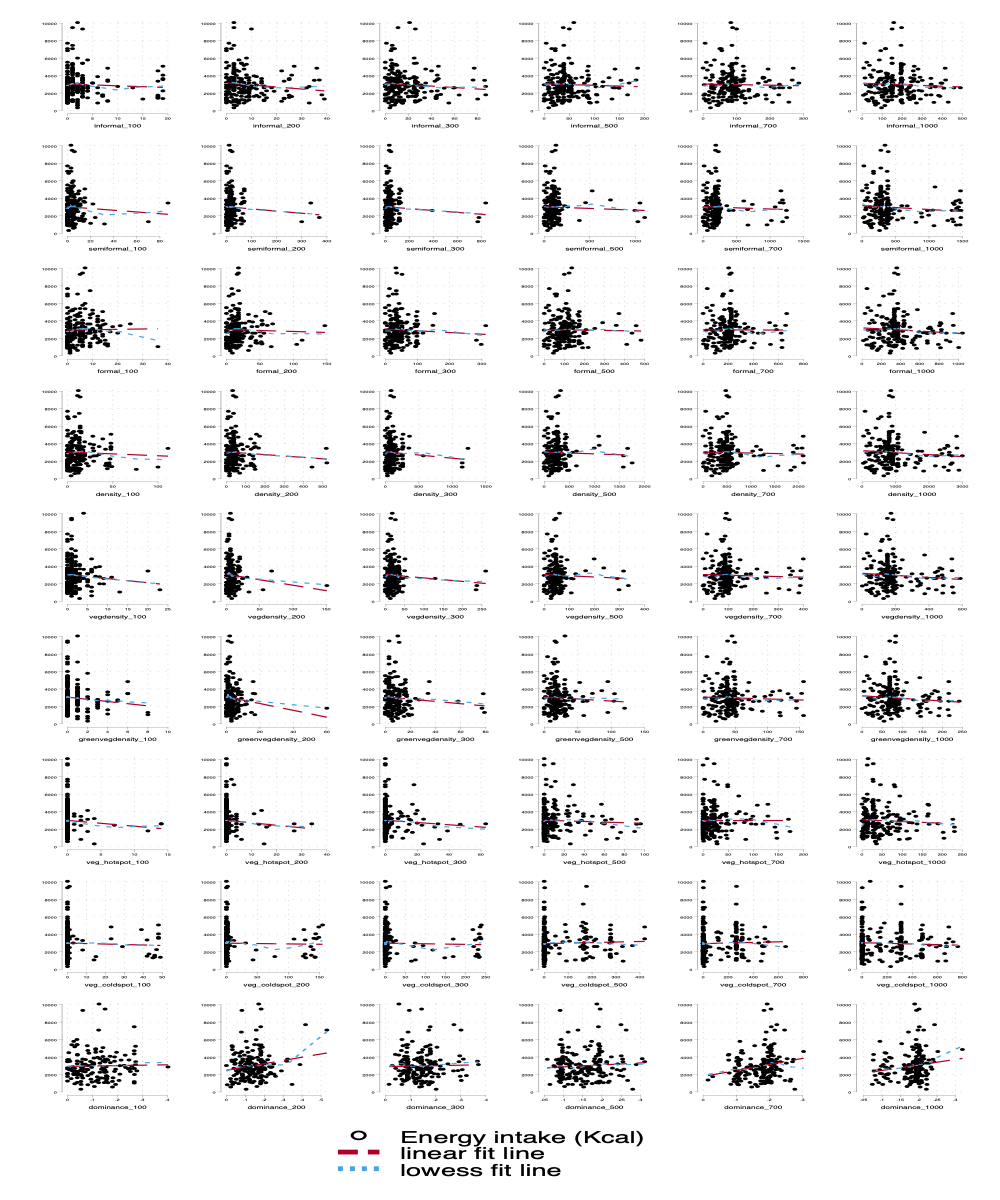


Supplemental Figure 4: Scatter plot with linear and lowess fit of various food environment metrics on waist to hip ratio


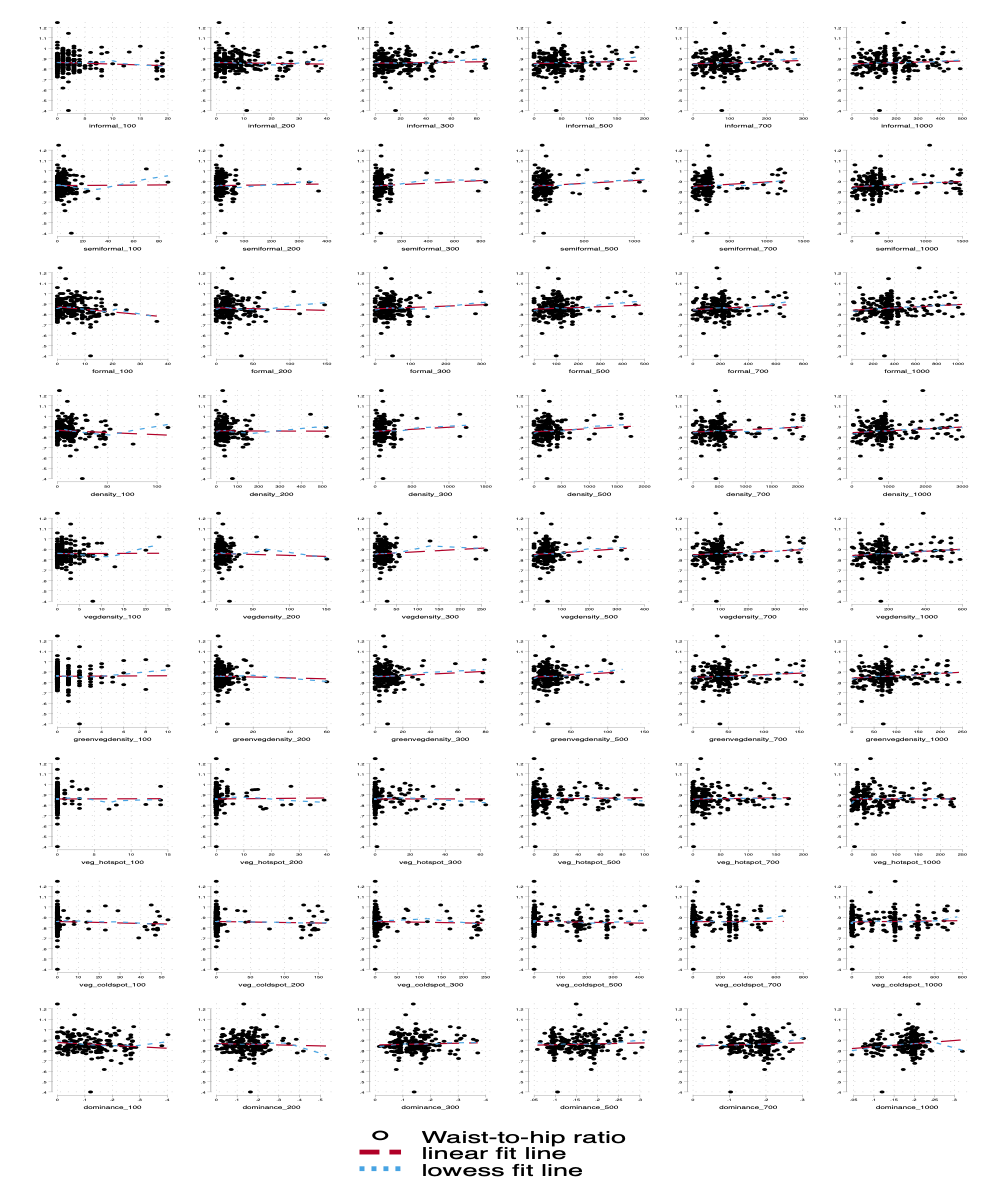


Supplemental Figure 5: Scatter plot with linear and lowess fit of various food environment metrics on body mass index.


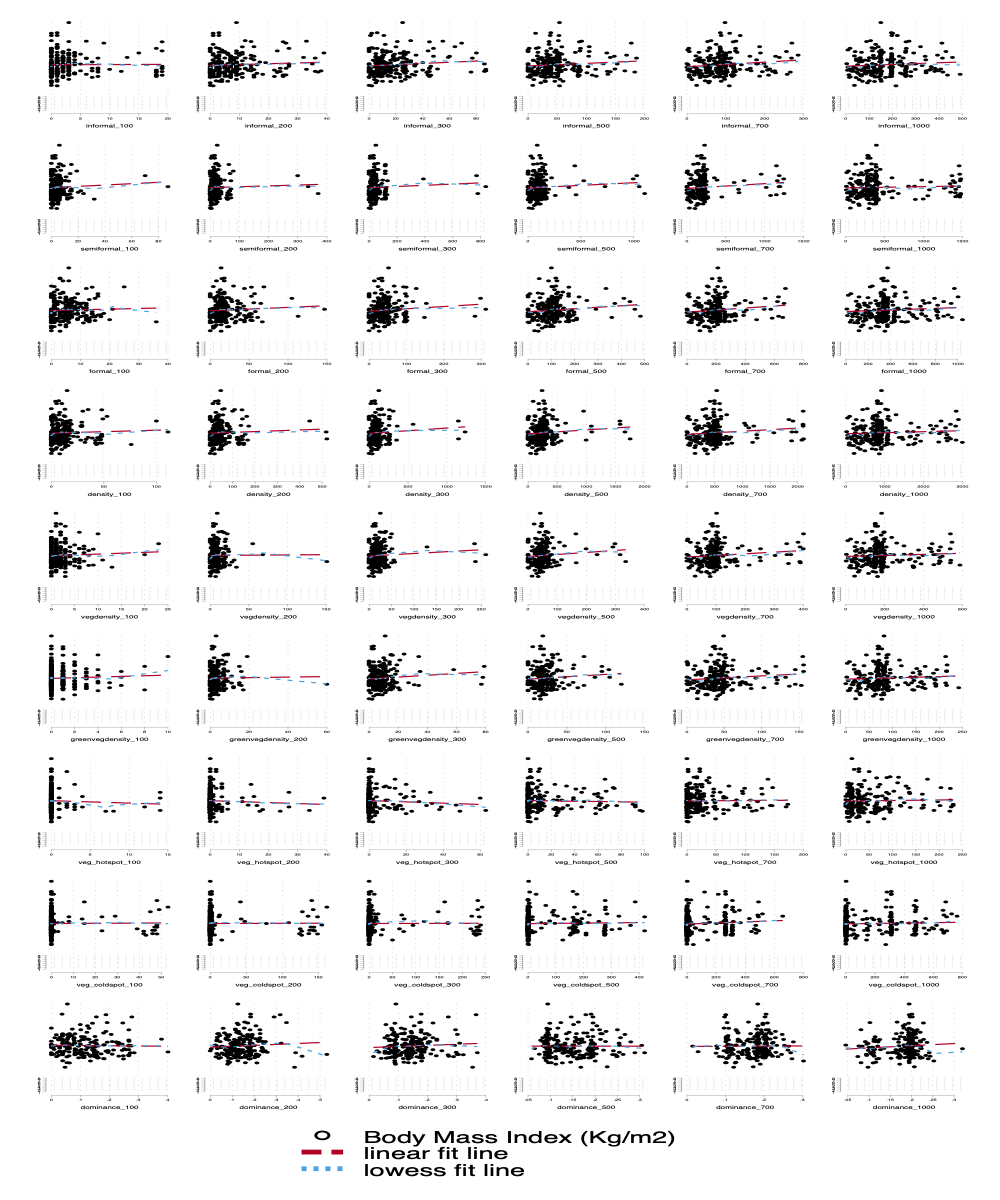


Supplemental Figure 6: Sensitivity analysis of dispersion by buffer sizes (0.1 and, 0.3km) on vegetable purchase in the last 7 days. All models adjusted for head of household status, assert quartiles, gender, age, house ownership, years since HIV diagnosis, education, presence of home garden and fridge.

Vegetable Purchase in the last 7 days

Vegetable Purchase variety in the last 7 days


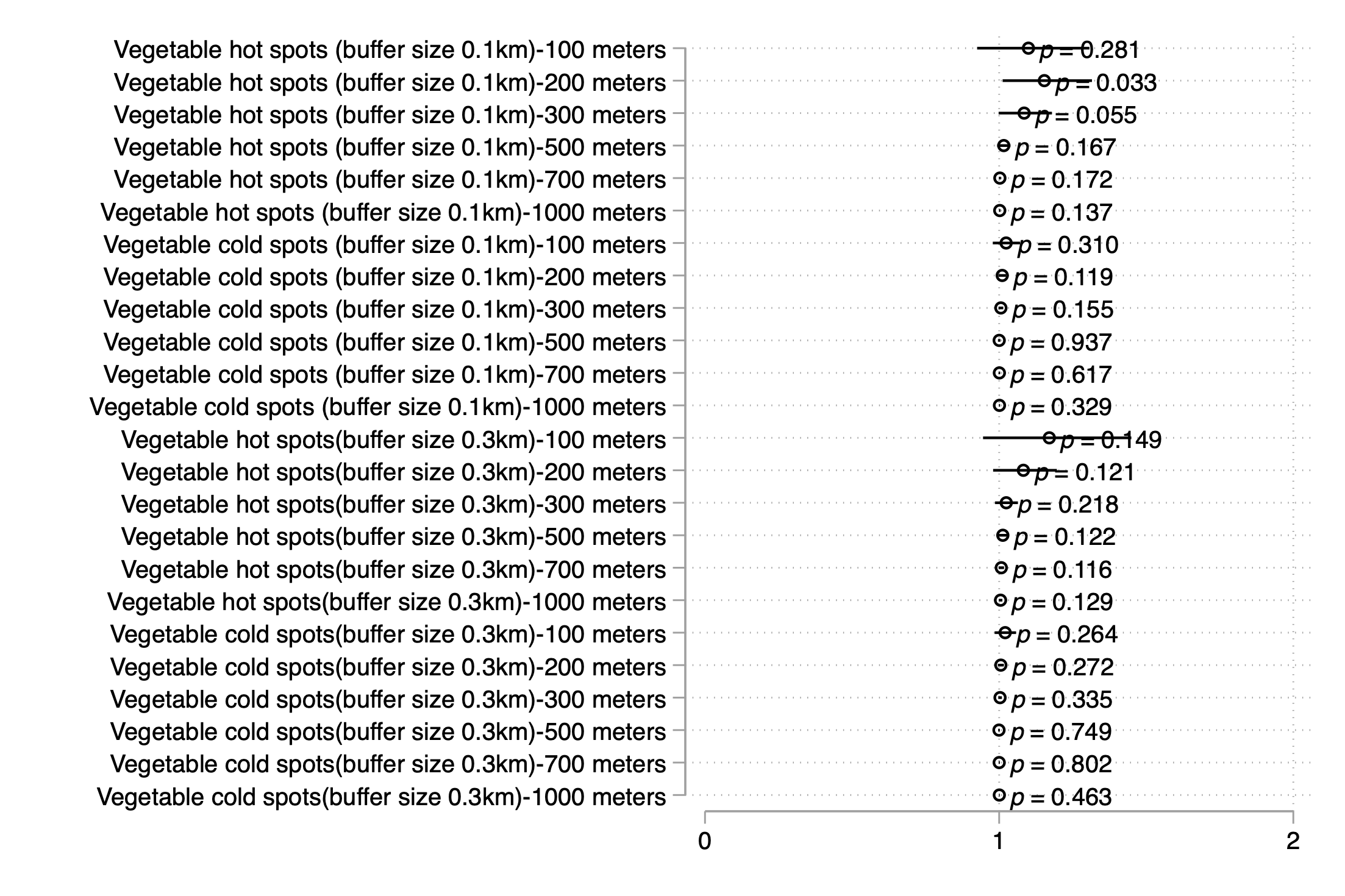

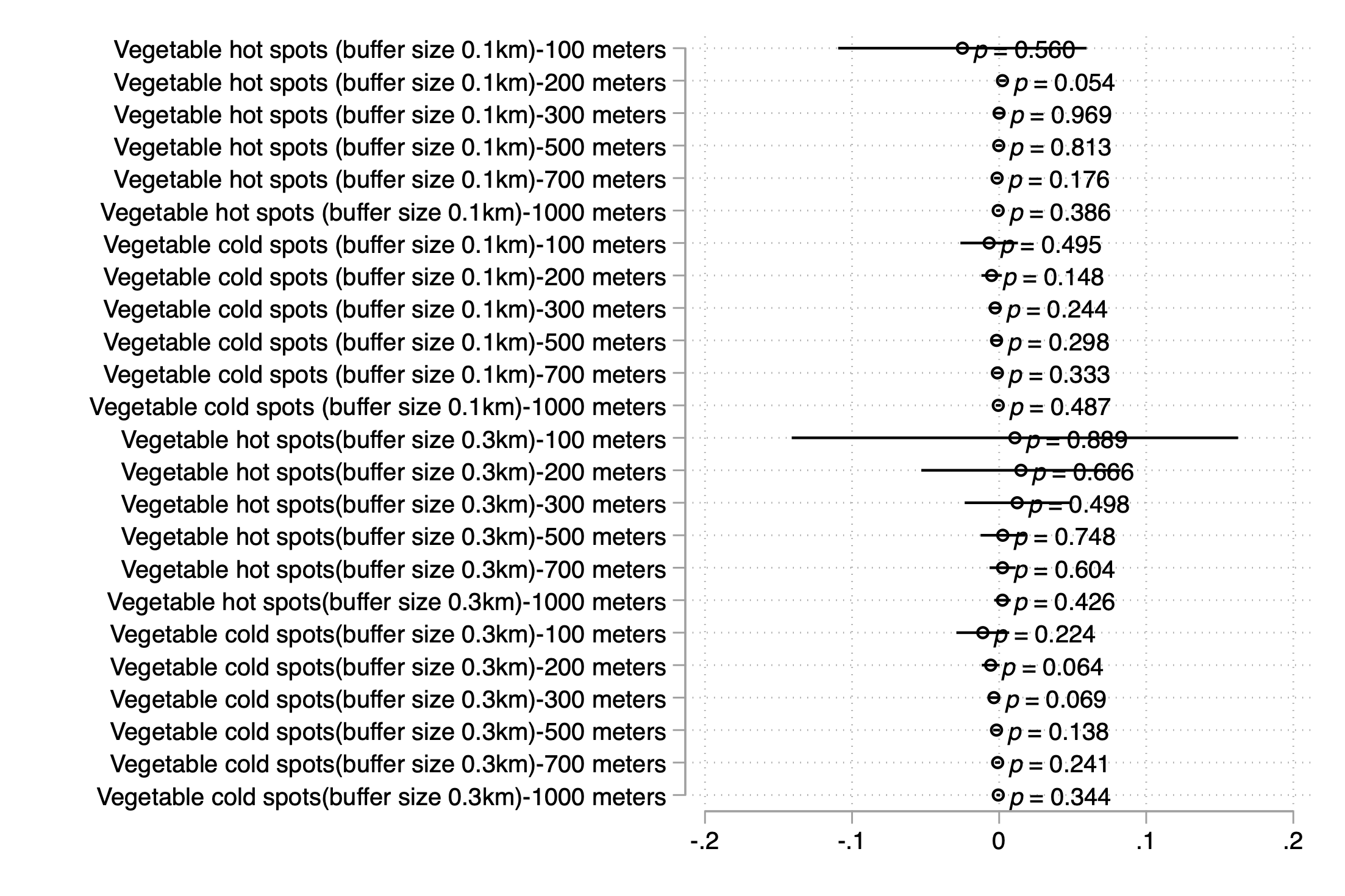


Supplemental Figure 7: Sensitivity analysis of dispersion by buffer sizes (0.1 and, 0.3km) on vegetable purchase in the last 7 days. All models adjusted for head of household status, assert quartiles, gender, age, house ownership, years since HIV diagnosis, education, presence of home garden and fridge.

Energy(Kcal)/Bodyweight (kg)

Energy (Kcal)


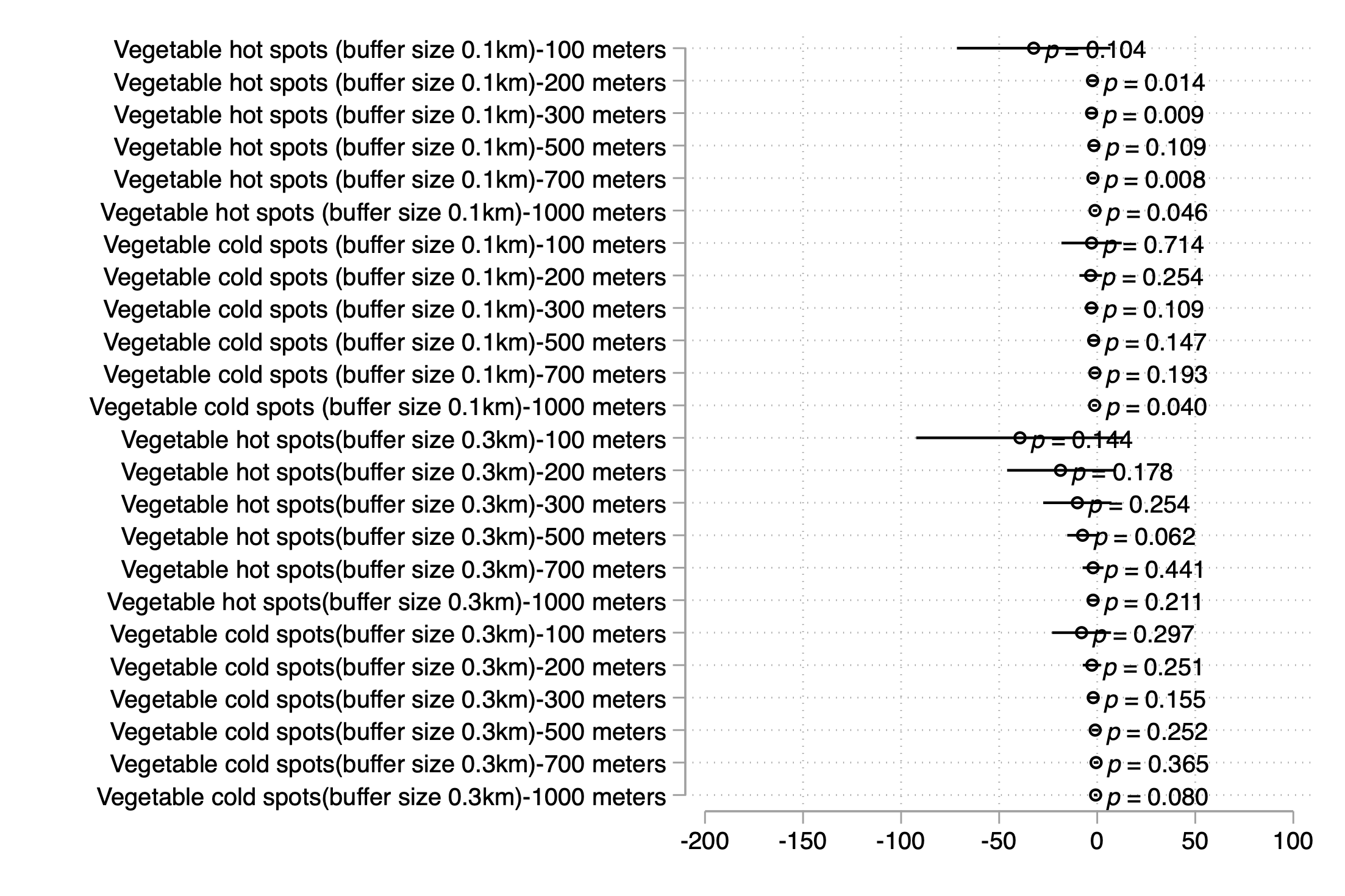

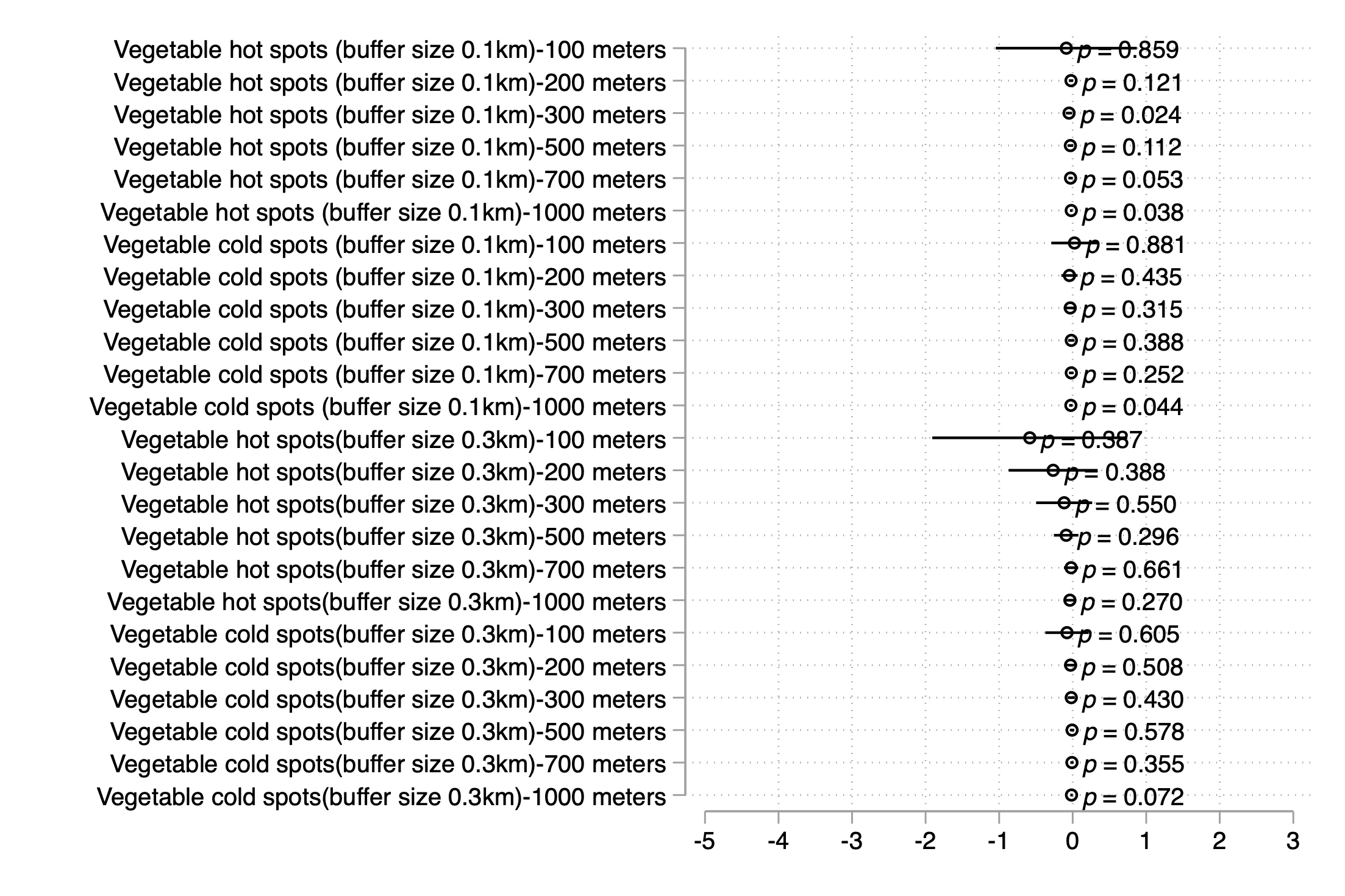


Supplemental Figure 8 Sensitivity analysis of dispersion by buffer sizes (0.1 and, 0.3km) on vegetable purchase in the last 7 days. All models adjusted for head of household status, assert quartiles, gender, age, house ownership, years since HIV diagnosis, education, presence of home garden and fridge.

Waist-to-Hip Ratios

Body Mass Index


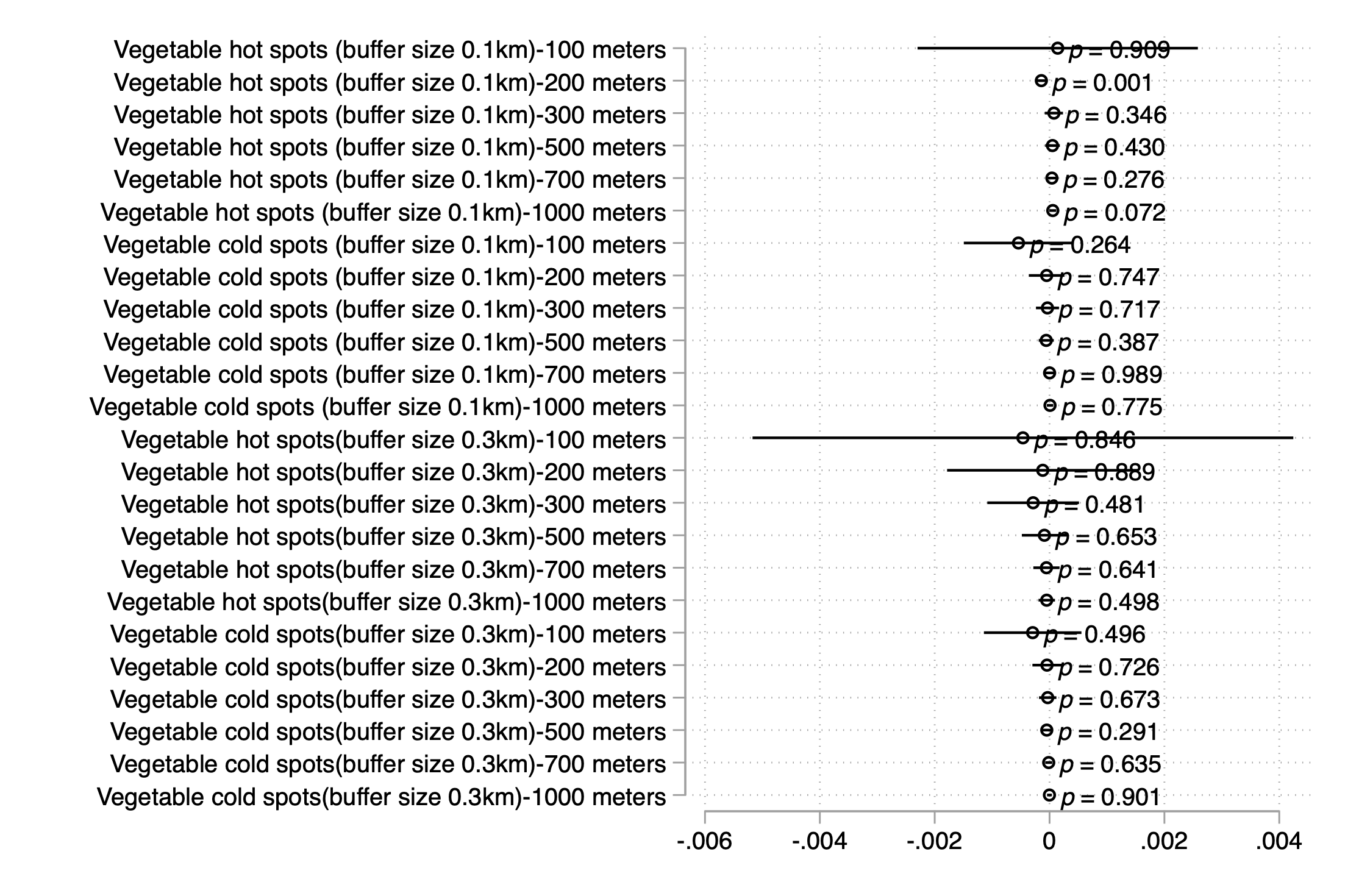

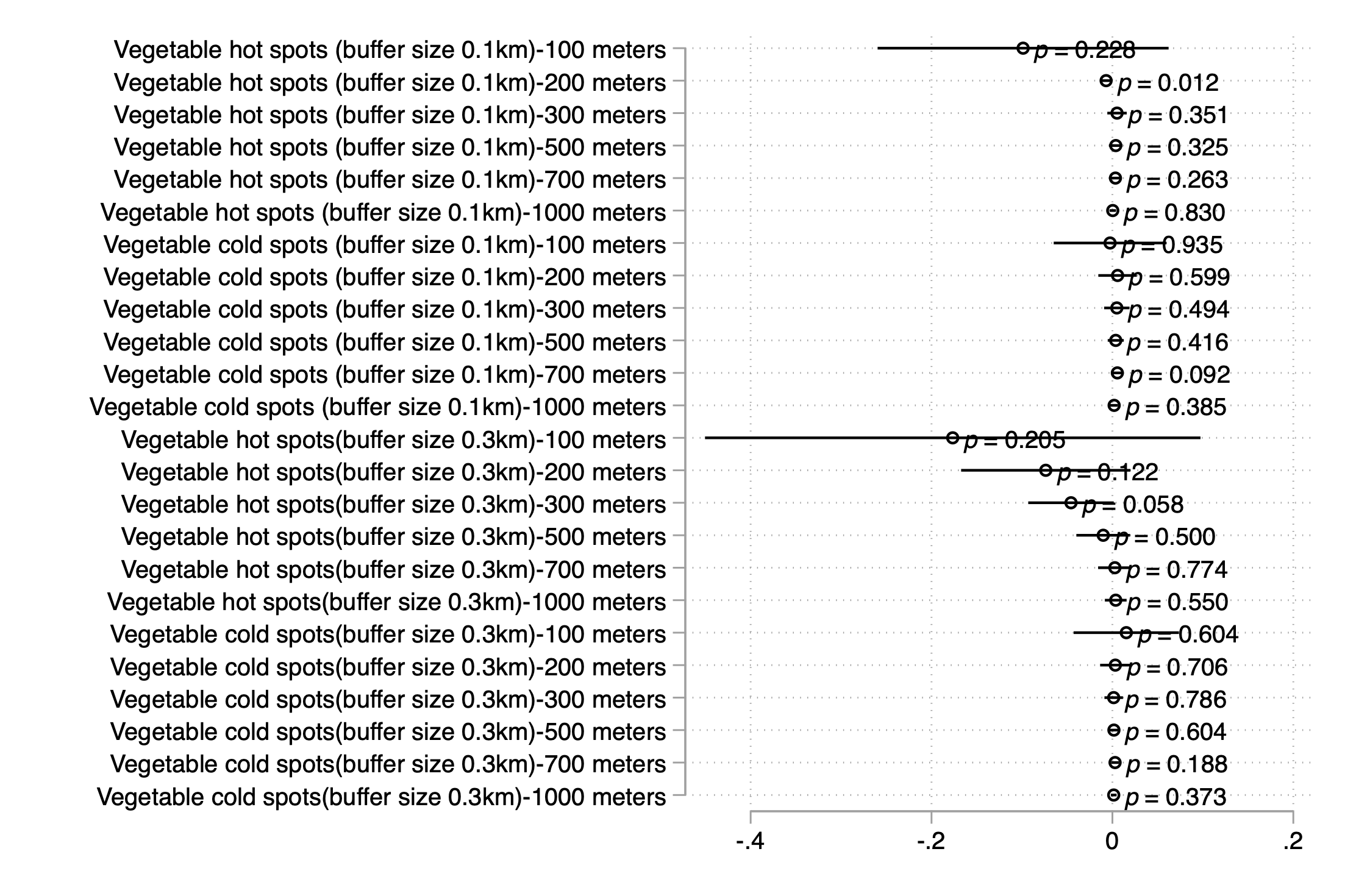


Supplemental Figure 9: Dominance metrics on outcomes. All models adjusted for head of household status, assert quartiles, gender, age, house ownership, years since HIV diagnosis, education, presence of home garden and fridge.

Vegetable Purchase in the last 7 days

Energy(Kcal)/Bodyweight (kg)

Vegetable Purchase variety in the last 7 days

Energy (Kcal)

Waist-to-Hip Ratios

Body Mass Index


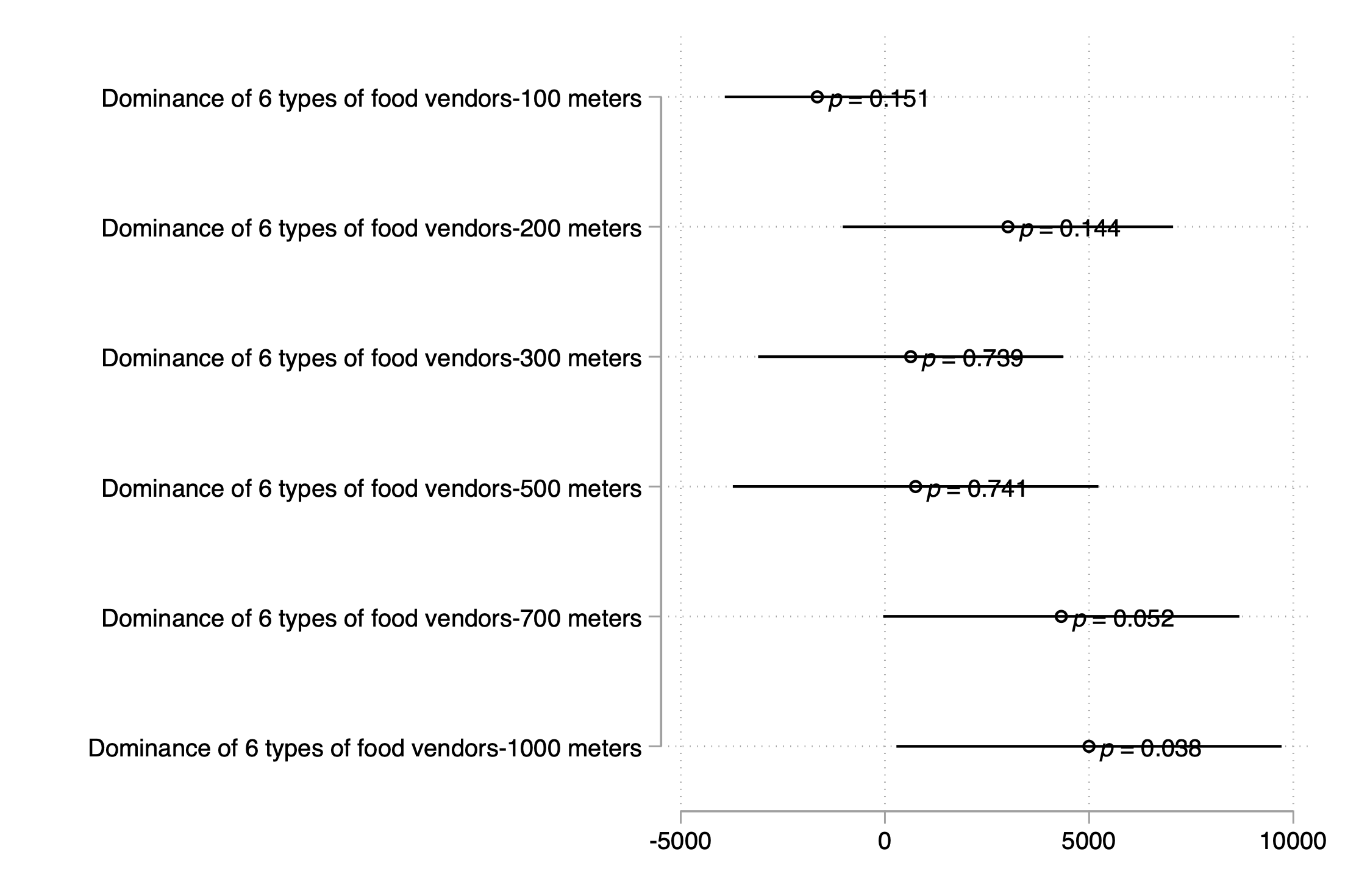

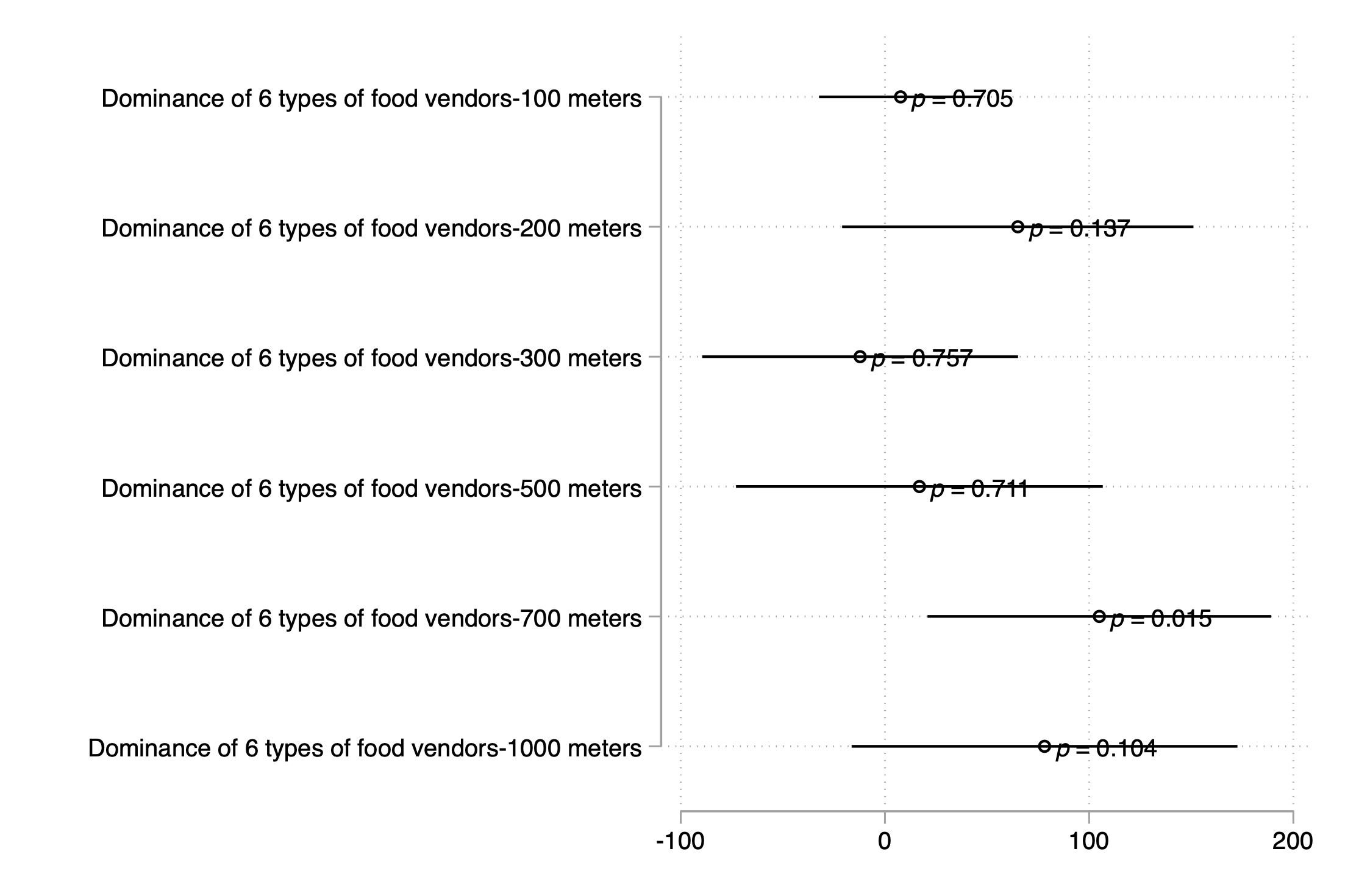

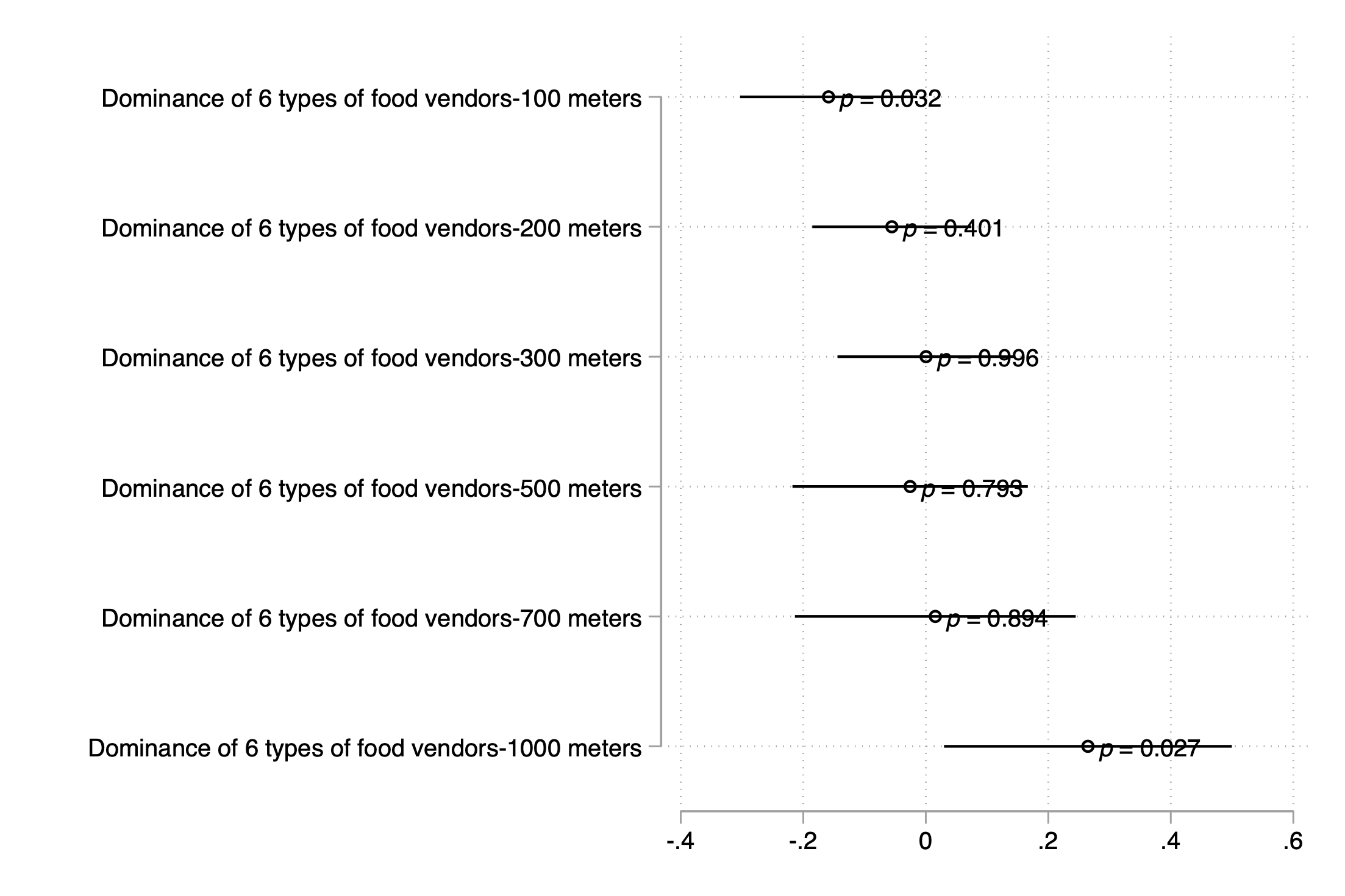

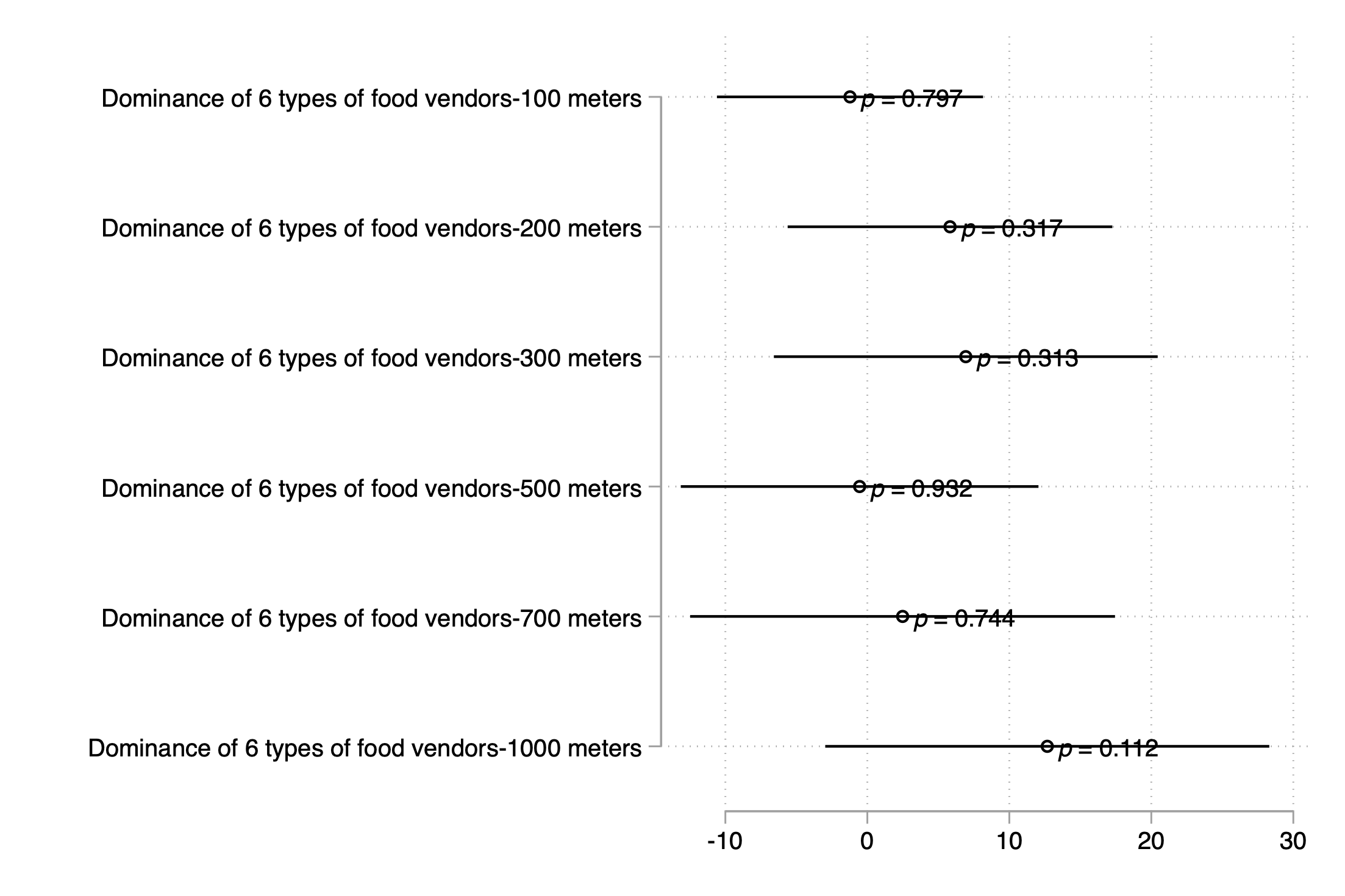

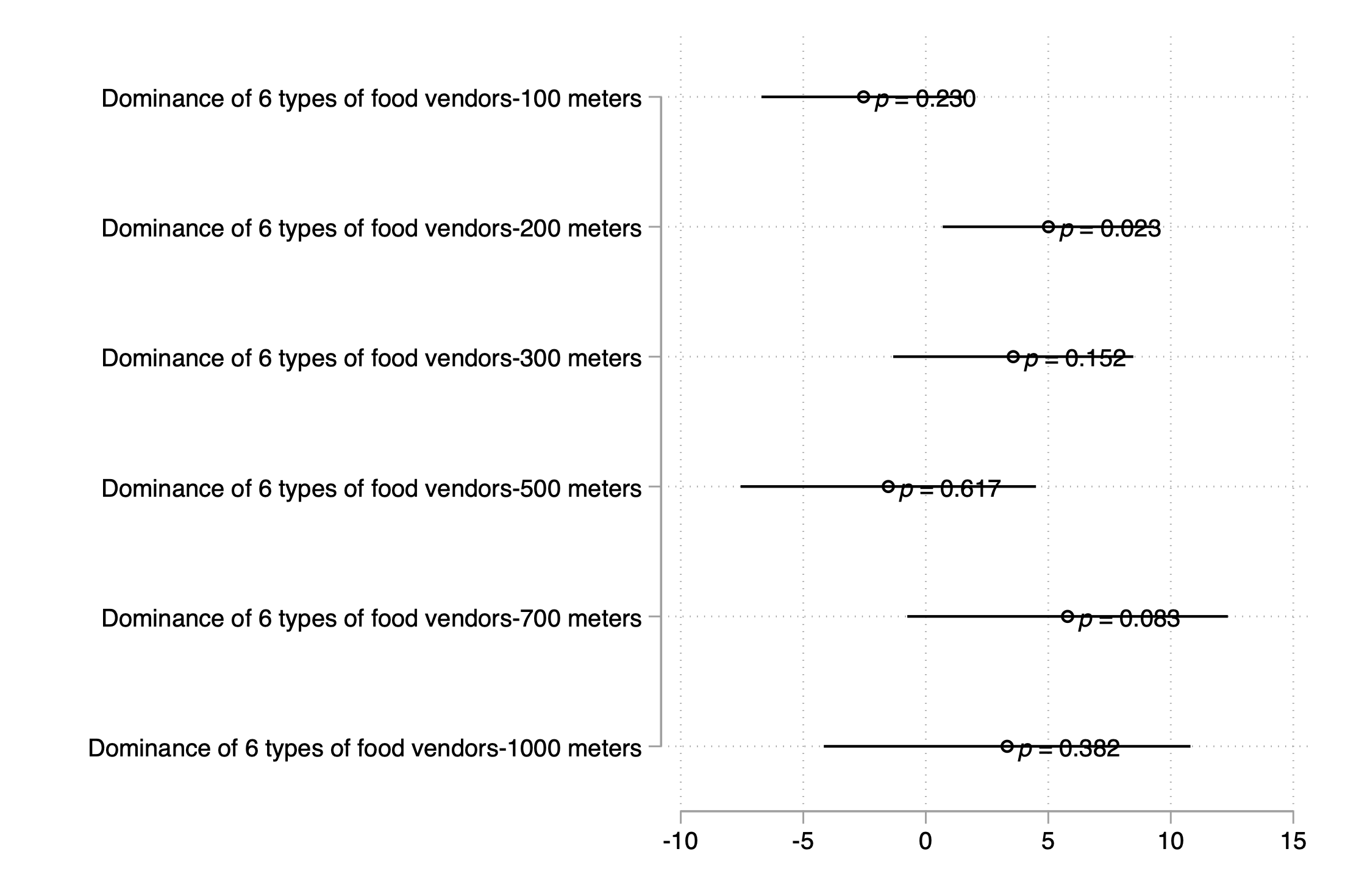

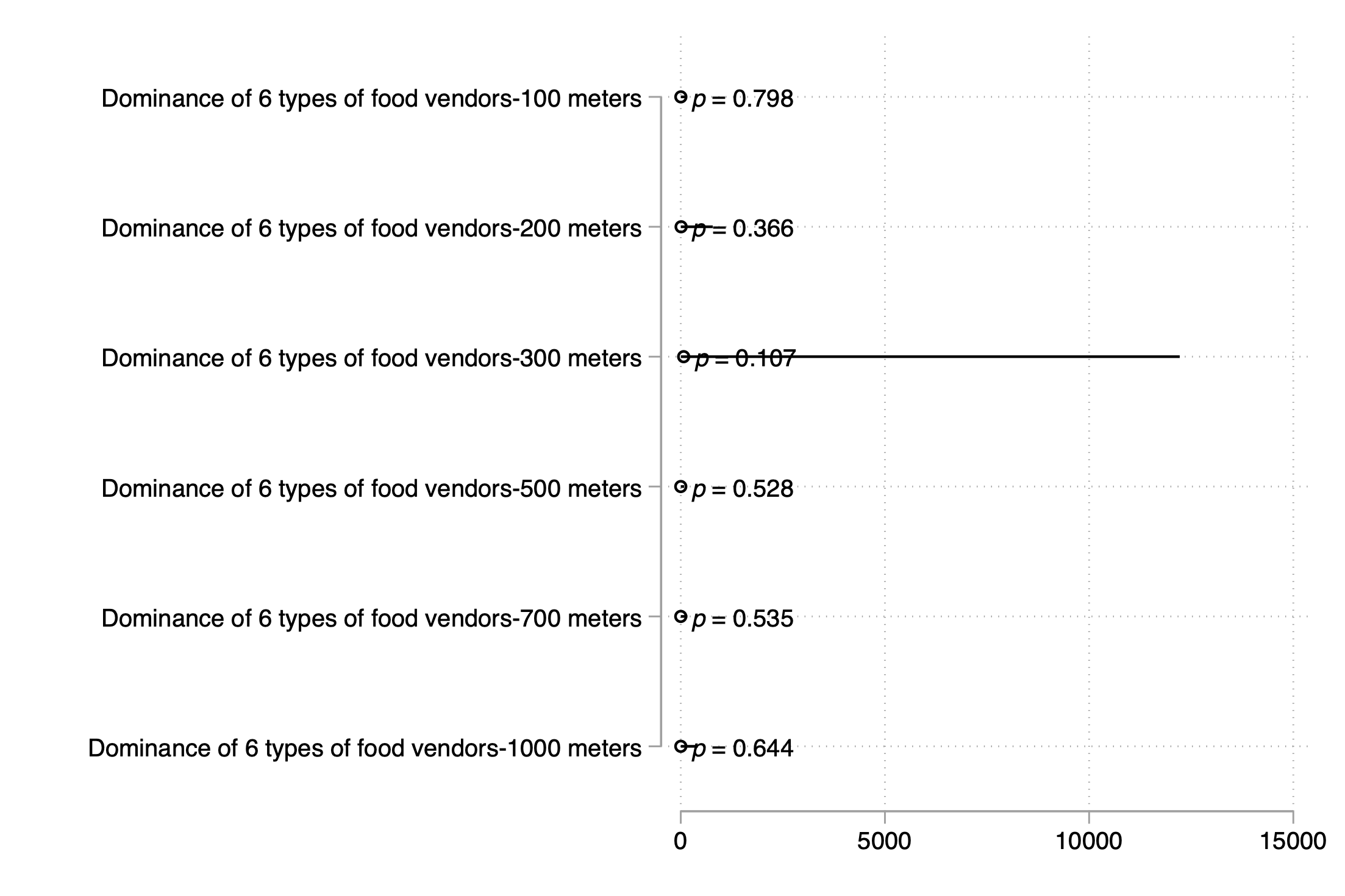

Supplement: Multimedia component 1 [file mmc1.docx]
